# Estimation of heterogeneity variance based on a generalized *Q* statistic in meta‐analysis of log‐odds‐ratio

**DOI:** 10.1002/jrsm.1647

**Published:** 2023-06-28

**Authors:** Elena Kulinskaya, David C. Hoaglin

**Affiliations:** ^1^ School of Computing Sciences University of East Anglia Norwich UK; ^2^ Department of Population and Quantitative Health Sciences UMass Chan Medical School Worcester Massachusetts USA

**Keywords:** effective‐sample‐size weights, heterogeneity, inverse‐variance weights, random effects

## Abstract

For estimation of heterogeneity variance $\tau^{2}$ in meta‐analysis of log‐odds‐ratio, we derive new mean‐ and median‐unbiased point estimators and new interval estimators based on a generalized Q statistic, $Q_{F}$, in which the weights depend on only the studies' effective sample sizes. We compare them with familiar estimators based on the inverse‐variance‐weights version of Q, $Q_{\mathrm{IV}}.$ In an extensive simulation, we studied the bias (including median bias) of the point estimators and the coverage (including left and right coverage error) of the confidence intervals. Most estimators add 0.5 to each cell of the 2×2 table when one cell contains a zero count; we include a version that always adds 0.5. The results show that: two of the new point estimators and two of the familiar point estimators are almost unbiased when the total sample size n≥250 and the probability in the Control arm ($p_{\mathrm{iC}}$) is 0.1, and when n≥100 and $p_{\mathrm{iC}}$ is 0.2 or 0.5; for $0.1\leq \tau^{2}\leq 1$, all estimators have negative bias for small to medium sample sizes, but for larger sample sizes some of the new median‐unbiased estimators are almost median‐unbiased; choices of interval estimators depend on values of parameters, but one of the new estimators is reasonable when $p_{\mathrm{iC}}=0.1$ and another, when $p_{\mathrm{iC}}=0.2$ or $p_{\mathrm{iC}}=0.5$; and lack of balance between left and right coverage errors for small n and/or $p_{\mathrm{iC}}$ implies that the available approximations for the distributions of $Q_{\mathrm{IV}}$ and $Q_{F}$ are accurate only for larger sample sizes.

**Highlights**

**What is already known**

- Use of inverse‐variance weights based on estimated variances in the conventional Q statistic makes it very difficult to approximate the null distribution of Q.
- Related moment‐based estimators of the heterogeneity variance ($\tau^{2}$), such as the DerSimonian‐Laird estimator, have considerable bias.

**What is new**

- We study estimation of $\tau^{2}$ based on $Q_{F}$, a generalized Q statistic with fixed effective‐sample‐size weights.
- For point estimation of $\tau^{2}$ we consider both mean‐unbiased and novel median‐unbiased estimators. The new estimators are based on the Farebrother approximation to the distribution of $Q_{F}$.
- In an extensive simulation study, we compared four new point estimators of $\tau^{2}$ and two new interval estimators with traditional estimators.
- We provide practical guidelines for choosing appropriate point and interval estimators for LOR.
- For median bias, which was not studied previously, an important finding is that the majority of the standard estimators have negative bias for larger sample sizes. This means that their median values of ${\hat{\tau }}^{2}$ are too low. Two new estimators consistently result in almost median‐unbiased estimation for moderate to large n.

**Potential impact for RSM readers outside the authors' field**

- We provide practical guidelines for choosing appropriate point and interval estimators of $\tau^{2}$ in meta‐analysis of log‐odds‐ratio.
- Some popular estimators of heterogeneity variance $\tau^{2}$ such as the DL and REML point estimators and PL intervals have unacceptable bias or coverage. We recommend instead new point and interval estimators of $\tau^{2}$ that use constant effective‐sample‐size weights. Some of these estimators are already implemented in *metafor*.

## 1 INTRODUCTION

As the measure of effect, meta‐analyses of binary outcomes from randomized trials most often use the odds ratio (OR), preferably analyzed as log‐odds‐ratio (LOR). The customary random‐effects analyses assess heterogeneity by using Cochran's Q statistic^1^ and estimate the between‐study variance, $\tau^{2}$, for use in inverse‐variance weights. The resulting weights, based on estimated variances, underlie various shortcomings of that approach. Thus, for assessing heterogeneity and estimating $\tau^{2}$, we have studied $Q_{F}$, a version of Cochran's Q statistic in which the weights involve only the studies' arm‐level sample sizes. $Q_{F}$ belongs to a class of generalized Q statistics, introduced by DerSimonian and Kacker,^2^ in which the $w_{i}$ are arbitrary positive constants.

Our initial motivation came from random‐effects meta‐analyses of mean difference (MD) and standardized mean difference (SMD)^3^ and, later, LOR,^4^ where a weighted mean whose weights involved only those effective sample sizes performed well in estimating the overall effect.

Further developments produced $Q_{F}$ and accurate approximations to its distribution function, for testing heterogeneity of MD,^5^ SMD,^5^ and three binary effect measures (LOR, log‐relative‐risk, and risk difference).^6^ Those studies also examined approximations for the customary Q statistic ($Q_{\mathrm{IV}}$) and investigated estimates for $\tau^{2}$ in MD and SMD.

In the present paper, we derive from $Q_{F}$ new point and interval estimators of $\tau^{2}$ for meta‐analysis of LOR. In particular, we study mean‐ and median‐unbiased estimators. Similar new estimators of $\tau^{2}$ are available in the procedure *rma* in *metafor*.^7^ However, the performance of these point and interval estimators of $\tau^{2}$ had not been evaluated by simulations. Therefore, we carried out an extensive simulation study of the bias of the point estimators and the coverage of the confidence intervals. For comparison we included familiar point and interval estimators of $\tau^{2}$.

Section 2 briefly reviews study‐level estimation of LOR. Section 3 reviews the generic random‐effects model and describes the Q statistic. Section 4 describes random‐effects models for LOR. Section 5 discusses approximations to the distributions of $Q_{F}$ and $Q_{\mathrm{IV}}$. Section 6 introduces new point and interval estimators of $\tau^{2}$ for LOR. Section 7 describes the simulation design, and Section 8 summarizes the results. Section 9 examines an example of meta‐analysis using LOR. Section 10 offers a summary and discussion. The Supporting Information provides further details on the interval estimators, the simulation results, and the example.

## 2 STUDY‐LEVEL ESTIMATION OF LOG‐ODDS‐RATIO

Analyses of log‐odds‐ratio usually adopt binomial distributions to model the numbers of events. In Study i (i=1,…,K), $X_{\mathrm{iT}}$ and $X_{\mathrm{iC}}$ denote the numbers of events in the $n_{\mathrm{iT}}$ subjects in the Treatment arm and the $n_{\mathrm{iC}}$ subjects in the Control arm. Thus, we treat $X_{\mathrm{iT}}$ and $X_{\mathrm{iC}}$ as independent binomial variables:

(1) $$X_{\mathrm{iT}}∼\mathrm{Bin}\left(n_{\mathrm{iT}},p_{\mathrm{iT}}\right)\;\text{and}\;X_{\mathrm{iC}}∼\mathrm{Bin}\left(n_{\mathrm{iC}},p_{\mathrm{iC}}\right).$$

The log‐odds‐ratio for Study i is

(2) $$\theta_{i}=\log_{e}\left(\frac{p_{\mathrm{iT}}\left(1-p_{\mathrm{iC}}\right)}{p_{\mathrm{iC}}\left(1-p_{\mathrm{iT}}\right)}\right)\;\text{estimated}\;\mathrm{by}\;{\hat{\theta }}_{i}=\log_{e}\left(\frac{{\overset{⌣}{p}}_{\mathrm{iT}}\left(1-{\overset{⌣}{p}}_{\mathrm{iC}}\right)}{{\overset{⌣}{p}}_{\mathrm{iC}}\left(1-{\overset{⌣}{p}}_{\mathrm{iT}}\right)}\right),$$

where ${\overset{⌣}{p}}_{\mathrm{ij}}$ is an estimate of $p_{\mathrm{ij}}$.

The customary estimators of $p_{\mathrm{iT}}$ and $p_{\mathrm{iC}}$ are ${\tilde{p}}_{\mathrm{ij}}=X_{\mathrm{ij}}/n_{\mathrm{ij}}$ (maximum likelihood). A reasonable alternative adds 0.5 to $X_{\mathrm{ij}}$ and adds 1 to $n_{\mathrm{ij}}$: ${\hat{p}}_{\mathrm{ij}}=\left(X_{\mathrm{ij}}+0.5\right)/\left(n_{\mathrm{ij}}+1\right)$ eliminates bias of order $1/n_{\mathrm{ij}}$ and provides the least biased estimator of log‐odds.^8^

As inputs, a two‐stage meta‐analysis uses estimates of the $\theta_{i}$ (${\hat{\theta }}_{i}$) and estimates of their variances (${\hat{v}}_{i}^{2}$). The (conditional, given $p_{\mathrm{ij}}$ and $n_{\mathrm{ij}}$) asymptotic variance of ${\hat{\theta }}_{i}$, derived by the delta method, is

(3) $$v_{i}^{2}=\mathrm{Var}\left({\hat{\theta }}_{i}\right)=\frac{1}{n_{\mathrm{iT}}p_{\mathrm{iT}}\left(1-p_{\mathrm{iT}}\right)}+\frac{1}{n_{\mathrm{iC}}p_{\mathrm{iC}}\left(1-p_{\mathrm{iC}}\right)},$$

estimated by substituting ${\tilde{p}}_{\mathrm{ij}}$ or ${\hat{p}}_{\mathrm{ij}}$ for $p_{\mathrm{ij}}$. The estimator of the variance using the ${\hat{p}}_{\mathrm{ij}}$ and $n_{\mathrm{ij}}+1$ instead of $n_{\mathrm{ij}}$ in (3) is unbiased in large samples, but Gart et al.^8^ note that it overestimates the variance for small sample sizes. As we explain in Section 4, the unconditional variance depends on the mechanism generating the $p_{\mathrm{iC}}$ and the $p_{\mathrm{iT}}$.

## 3 RANDOM‐EFFECTS MODEL AND THE Q STATISTIC.

In the most widely used method for two‐stage meta‐analysis,^9^ Cochran's Q statistic serves as the basis for testing heterogeneity and estimating $\tau^{2}$, for use in the inverse‐variance weights, ${\hat{w}}_{i}=1/\left({\hat{v}}_{i}^{2}+{\hat{\tau }}^{2}\right)$. Q is a weighted sum of the squared deviations of the estimated effects ${\hat{\theta }}_{i}$ from their weighted mean ${\bar{\theta }}_{w}=\sum w_{i}{\hat{\theta }}_{i}/\sum w_{i}$:

(4) $$Q=\sum w_{i}{\left({\hat{\theta }}_{i}-{\bar{\theta }}_{w}\right)}^{2}.$$

In calculating Q, an estimate of $\tau^{2}$ is not yet available, so $w_{i}$ is simply $1/{\hat{v}}_{i}^{2}$, the reciprocal of the *estimated* variance of ${\hat{\theta }}_{i}$ (as in Cochran^1^). We denote the result by $Q_{\mathrm{IV}}$.

The process underlying the DerSimonian‐Laird^9^ estimator of $\tau^{2}$ (${\hat{\tau }}_{\mathrm{DL}}^{2}$) derives the expected value of $Q_{\mathrm{IV}}$ and rearranges the resulting expression to obtain $\tau^{2}$ in terms of $E\left(Q_{\mathrm{IV}}\right)$ and the $v_{i}^{2}=\mathrm{Var}\left({\hat{\theta }}_{i}\right)$. Substituting the observed value of $Q_{\mathrm{IV}}$ for $E\left(Q_{\mathrm{IV}}\right)$ and the ${\hat{v}}_{i}^{2}$ for the $v_{i}^{2}$ then yields ${\hat{\tau }}_{\mathrm{DL}}^{2}$. The convenient step of plugging in the ${\hat{v}}_{i}^{2}$, however, lacks justification; it inderlies the documented shortcomings of the DL method.

As an alternative, we base estimates of $\tau^{2}$ on $Q_{F}$, studied by Kulinskaya et al.,^5^ in which $w_{i}={\tilde{n}}_{i}=n_{\mathrm{iC}}n_{\mathrm{iT}}/n_{i}$, the effective sample size in Study i ($n_{i}=n_{\mathrm{iC}}+n_{\mathrm{iT}}$).

In studying properties of estimators under the random‐effects model, we start by taking as given the observed study‐level effects ${\hat{\theta }}_{i}$; that is, we condition on those values. The model includes a distribution for the true $\theta_{i}$, conventionally $\theta_{i}∼N\left(\theta ,\tau^{2}\right)$. Taking expectations with respect to that model yields unconditional estimators, for comparison with the conditional ones.

The random‐effects model assumes that the ${\hat{\theta }}_{i}$ are unbiased estimators of the $\theta_{i}$ and that the $v_{i}^{2}=\mathrm{Var}\left({\hat{\theta }}_{i}|\theta_{i}\right)$ are the corresponding variances (i.e., the true conditional variances).

To develop estimators of $\tau^{2}$ based on $Q_{F}$, we define $W=\sum w_{i}$, $q_{i}=w_{i}/W$, and $\Theta_{i}={\hat{\theta }}_{i}-\theta$. In this notation, and expanding ${\bar{\theta }}_{w}$, Equation (4) can be written as

(5) Q=W∑qi1−qiΘi2−∑i=jqiqjΘiΘj.

We distinguish between the conditional distribution of Q (given the $\theta_{i}$) and the unconditional distribution, and the respective moments of $\Theta_{i}$. For instance, the conditional second moment of $\Theta_{i}$, denoted by $M_{2i}^{c}$, is $v_{i}^{2}$; and the unconditional second moment, denoted by $M_{2i}$, is $E\left(\Theta_{i}^{2}\right)=\mathrm{Var}\left({\hat{\theta }}_{i}\right)=E\left(v_{i}^{2}\right)+\tau^{2}$.

Under the REM, it is straightforward to obtain the first moment of $Q_{F}$ as

(6) $$E\left(Q_{F}\right)=W\left[\sum q_{i}\left(1-q_{i}\right)E\left(\Theta_{i}^{2}\right)\right]=W\left[\sum q_{i}\left(1-q_{i}\right)\left(E\left(v_{i}^{2}\right)+\tau^{2}\right)\right].$$

This expression is similar to Equation (4) in DerSimonian and Kacker^2^; they use $v_{i}^{2}+\tau^{2}$ instead of the unconditional variance $E\left(v_{i}^{2}\right)+\tau^{2}$.

Our simulations yield an exact calculation of conditional central moments of LOR, following the implementation of Kulinskaya and Dollinger.^10^

## 4 RANDOM‐EFFECTS META‐ANALYSIS OF LOG‐ODDS‐RATIO

The standard REM for LOR assumes that $\text{logit}\left(p_{\mathrm{iT}}\right)=\text{logit}\left(p_{\mathrm{iC}}\right)+\theta_{i}$ for $\theta_{i}∼N\left(\theta ,\tau^{2}\right)$. The intercept $\alpha_{i}=\text{logit}\left(p_{\mathrm{iC}}\right)$ may also be random. Further, $p_{\mathrm{iC}}$ and $p_{\mathrm{iT}}$ may be correlated. Equation (3) gives the conditional variance of ${\hat{\theta }}_{i}$. The full (unconditional) variance of ${\hat{\theta }}_{i}$ depends on the generation mechanism for the $p_{\mathrm{iC}}$ and was derived in Kulinskaya et al.^11^

Conventionally, the $p_{\mathrm{iC}}$ are assumed to be fixed. Then

(7) $$E\left(v_{i}^{2}\right)=\frac{1}{n_{\mathrm{iT}}{\overset{⌣}{p}}_{\mathrm{iT}}\left(1-{\overset{⌣}{p}}_{\mathrm{iT}}\right)}+\frac{1}{n_{\mathrm{iC}}p_{\mathrm{iC}}\left(1-p_{\mathrm{iC}}\right)}+\tau^{2}\left(1+\frac{1}{2n_{\mathrm{iT}}}\left({\left[{\overset{⌣}{p}}_{\mathrm{iT}}\left(1-{\overset{⌣}{p}}_{\mathrm{iT}}\right)\right]}^{-1}-2\right)\right),$$

where ${\overset{⌣}{p}}_{\mathrm{iT}}=\text{expit}\left(\alpha_{i}+\theta \right)$. For use in $Q_{F}$, this unconditional variance can be estimated by substituting ${\hat{p}}_{\mathrm{iC}}$ for $p_{\mathrm{iC}}$ and ${\bar{p}}_{\mathrm{iT}}=\text{expit}\left({\hat{\alpha }}_{i}+\hat{\theta }\right)$ for ${\overset{⌣}{p}}_{\mathrm{iT}}$, where ${\hat{\alpha }}_{i}=\text{logit}\left({\hat{p}}_{\mathrm{iC}}\right)$ and $\hat{\theta }$ is the estimated LOR. We refer to the estimate ${\bar{p}}_{\mathrm{iT}}$ of $p_{\mathrm{iT}}$ as model‐based. Alternatively, a naïve estimate uses ${\hat{p}}_{\mathrm{iT}}$. Such a naïve estimate has the advantage that it maintains the variance inflation of $E\left(v_{i}^{2}\right)$ in comparison with $v_{i}^{2}$ (using $v_{i}^{2}$ from Equation (3)). Whenever ${\hat{p}}_{\mathrm{ij}}$ is used in Equation (7), $n_{\mathrm{ij}}+1$ replaces $n_{\mathrm{ij}}$.

We use these results in Sections 5 and 6.

## 5 APPROXIMATIONS TO THE DISTRIBUTIONS OF $Q_{F}$ AND $Q_{\mathrm{IV}}$

Our new interval estimators of $\tau^{2}$ (Section 6.2) involve the cumulative distribution function of $Q_{F}$. For LOR, $Q_{F}$ is a quadratic form in asymptotically normal variables. The Farebrother algorithm,^12^ applicable for quadratic forms in normal variables, provides a satisfactory approximation to the cdf for larger sample sizes (n≥100), though it may not behave well for small n.^6^ To apply it, we plug in estimated variances. As in Reference [13], we denote the Farebrother approximation for Q with effective‐sample‐size weights by F SSW. We further distinguish between a “model‐based” version and a “naïve” version of F SSW, according to whether we use ${\bar{p}}_{\mathrm{iT}}$ or ${\hat{p}}_{\mathrm{iT}}$ in Equation (7).

The null distribution of $Q_{\mathrm{IV}}$ is usually approximated by the chi‐square distribution with K−1 degrees of freedom. For LOR, as also for both MD and SMD, this approximation is not accurate for small sample sizes.^14^ For LOR, Kulinskaya and Dollinger^10^ provided an improved approximation to the null distribution of $Q_{\mathrm{IV}}$ based on fitting two moments of the gamma distribution; we denote this approximation by KD. For comparison, we study point and interval estimators of $\tau^{2}$ based on the chi‐square and KD approximations.

## 6 POINT AND INTERVAL ESTIMATORS OF $\tau^{2}$ FOR LOR

### 6.1 Point estimators

The unconditional variance of $\hat{\theta }$ in the customary fixed‐intercept model, Equation (7), can be written as a sum of two terms,

(8) $$M_{2i}=E\left(\mathrm{Var}\left({\hat{\theta }}_{i}|p_{\mathrm{ij}},n_{\mathrm{ij}}\right)\right)+\tau^{2}\left(1+\frac{1}{2n_{\mathrm{iT}}}\left({\left[{\overset{⌣}{p}}_{\mathrm{iT}}\left(1-{\overset{⌣}{p}}_{\mathrm{iT}}\right)\right]}^{-1}-2\right)\right),$$

where $E\left(\mathrm{Var}\left({\hat{\theta }}_{i}|p_{\mathrm{ij}},n_{\mathrm{ij}}\right)\right)={\left[n_{\mathrm{iT}}{\overset{⌣}{p}}_{\mathrm{iT}}\left(1-{\overset{⌣}{p}}_{\mathrm{iT}}\right)\right]}^{-1}+{\left[n_{\mathrm{iC}}p_{\mathrm{iC}}\left(1-p_{\mathrm{iC}}\right)\right]}^{-1}$, $p_{\mathrm{iC}}=\text{expit}\left(\alpha_{i}\right)$, and ${\overset{⌣}{p}}_{\mathrm{iT}}=\text{expit}\left(\alpha_{i}+\theta \right)$. Rearranging the terms in Equation (6) with $E\left(\Theta_{i}^{2}\right)=M_{2i}$ and replacing $p_{\mathrm{iC}}$ and ${\overset{⌣}{p}}_{\mathrm{iT}}$ with ${\hat{p}}_{\mathrm{iC}}$ and ${\hat{p}}_{\mathrm{iT}}$ (or ${\bar{p}}_{\mathrm{iT}}$) give the naïve (or model‐based) moment estimator of $\tau^{2}$.

(9) $${\hat{\tau }}_{U}^{2}=\frac{Q/W-\sum q_{i}\left(1-q_{i}\right){\hat{v}}_{i}^{2}}{\sum q_{i}\left(1-q_{i}\right)C_{i}},$$

where ${\hat{v}}_{i}^{2}={\left[n_{\mathrm{iT}}{\hat{p}}_{\mathrm{iT}}\left(1-{\hat{p}}_{\mathrm{iT}}\right)\right]}^{-1}+{\left[n_{\mathrm{iC}}{\hat{p}}_{\mathrm{iC}}\left(1-{\hat{p}}_{\mathrm{iC}}\right)\right]}^{-1}$ and $C_{i}=1+\frac{1}{2n_{\mathrm{iT}}}\left({\left[{\hat{p}}_{\mathrm{iT}}\left(1-{\hat{p}}_{\mathrm{iT}}\right)\right]}^{-1}-2\right)$. DerSimonian and Kacker^2^ obtain a similar result; they use the conditional estimate, ${\hat{v}}_{i}^{2}$, instead of the unconditional estimate, $\hat{E\left(v_{i}^{2}\right)}$, obtaining

(10) $${\hat{\tau }}_{M}^{2}=\frac{Q/W-\sum q_{i}\left(1-q_{i}\right){\hat{v}}_{i}^{2}}{\sum q_{i}\left(1-q_{i}\right)}.$$

We study both estimators with effective‐sample‐size weights. With the conditional estimated variances in Equation (10), we denote the estimator by SSC (for “Sample Sizes Conditional”); with the unconditional estimated variances, as in Equation (9), it is SSU (for “Sample Sizes Unconditional”). These estimators differ by a term of order $O\left(1/n_{i}\right)$ and will be very similar for large sample sizes.

The estimators ${\hat{\tau }}_{U}^{2}$ and ${\hat{\tau }}_{M}^{2}$ arose from setting the observed value of Q equal to its expected value and solving for $\tau^{2}$. Instead of the expected value, one could use the median of the distribution of Q given $\tau^{2}$.^15^, ^16^, ^17^ If the true (or approximate) cumulative distribution function is $F\left(\cdot |\tau^{2}\right)$, a point estimator of $\tau^{2}$ can be found as

(11) $${\hat{\tau }}_{\mathrm{med}}^{2}=\max\left(0,\;\left\{\tau^{2}:F\left(Q|\tau^{2}\right)=0.5\right\}\right).$$

In the Farebrother approximation to the distribution of $Q_{F}$ (Section 5), one can use either the conditional estimated variances or the unconditional estimated variances. We denote the resulting estimators by SMC and SMU (“Sample sizes Median (Un)Conditional”), respectively.

Choosing between the “model‐based” and the “naïve” estimate of $p_{\mathrm{iT}}$ in $M_{2i}$ (8) yields “model‐based” and “naïve” versions of SSU and SMU: SSU model or SSU naïve and SMU model or SMU naïve.

The SSC and SMC estimators can be obtained from the procedure *rma* in *metafor* ^7^ by choosing as the method “GENQ” or “GENQM,” respectively, and specifying nbar weights.

For comparison, our simulations (Section 7) include four estimators that use inverse‐variance weights: DerSimonian‐Laird^9^ (DL), restricted maximum‐likelihood (REML), Mandel‐Paule^18^ (MP), and an estimator (KD) based on the work of Kulinskaya and Dollinger^10^ and discussed by Bakbergenuly et al.^4^ KD uses an improved non‐null first moment of Q and has better performance than most other estimators of $\tau^{2}$. In their review of methods for estimating the between‐study variance, Veroniki et al.^19^ explain that DL is (by default) the most widely used, and they conclude that both REML and MP are better.

A perennial question involves whether analysts should add 1/2 to each of $X_{\mathrm{iT}},\;X_{\mathrm{iC}},\;n_{\mathrm{iT}}-X_{\mathrm{iT}},\;n_{\mathrm{iC}}-X_{\mathrm{iC}}$ only when one of them is zero, or in all studies. This is equivalent to using $\tilde{p}$ (whenever possible) or $\hat{p}$ (always), respectively, when estimating $\theta_{i}$ and $v_{i}^{2}$. To obtain evidence on this issue, we included the corresponding two versions, “only” and “always,” of DL, REML, MP, SSC, and SMC (we follow the prevalent practice of omitting “double‐zero” studies, in which two of those cell counts are zero).

Table 1 gives the full list of point estimators.

**TABLE 1:** Point and interval estimators of $\tau^{2}$ in the simulations.

Estimator	Description	Add 1/2	
		“Only”	“Always”
Point estimators			
DL	DerSimonian‐Laird,^9^ a moment estimator based on $\chi^{2}$ approximation to distribution of $Q_{\mathrm{IV}}$	x	x
REML	Restricted Maximum Likelihood	x	x
MP	Mandel‐Paule,^18^ a moment estimator based on $\chi^{2}$ approximation to distribution of $Q_{\mathrm{IV}}$	x	x
KD	Kulinskaya‐Dollinger,^10^ Bakbergenuly et al.^4^ a moment estimator based on improved Gamma approximation to $Q_{\mathrm{IV}}$		x
SSC	Equation (10), effective‐sample‐size weights, conditional variance (3) of $\hat{\theta }$	x	x
SSU model	Equation (9), effective‐sample‐size weights, model‐based estimate of $p_{\mathrm{iT}}$ in unconditional variance (7) of $\hat{\theta }$		x
SSU naïve	Equation (9), effective‐sample‐size weights, naïve estimate of $p_{\mathrm{iT}}$ in unconditional variance (7) of $\hat{\theta }$		x
SMC	Median‐unbiased, Equation (11), effective‐sample‐size weights, conditional variance (3) of $\hat{\theta }$	x	x
SMU model	Median‐unbiased, Equation (11), effective‐sample‐size weights, model‐based estimate of $p_{\mathrm{iT}}$ in unconditional variance (7) of $\hat{\theta }$		x
SMU naïve	Median‐unbiased, Equation (11), effective‐sample‐size weights, naïve estimate of $p_{\mathrm{iT}}$ in unconditional variance (7) of $\hat{\theta }$		x
Confidence intervals			
QP	Q‐profile, Viechtbauer,^20^ Appendix S1.2	x	x
PL	Profile Likelihood, Hardy & Thompson,^21^ Appendix S1.1	x	x
KD	Kulinskaya–Dollinger,^10^ Bakbergenuly et al.,^4^ Appendix S1.3, profiled improved Gamma approximation to distribution of $Q_{\mathrm{IV}}$		x
FPC	Farebrother Profile, that is, profiled Farebrother approximation to distribution of $Q_{F}$, effective‐sample‐size weights, conditional variance (3) of $\hat{\theta }$	x	x
FPU model	Farebrother Profile, effective‐sample‐size weights, model‐based estimate of $p_{\mathrm{iT}}$ in unconditional variance (7) of $\hat{\theta }$		x
FPU naïve	Farebrother Profile, effective‐sample‐size weights, naïve estimate of $p_{\mathrm{iT}}$ in unconditional variance (7) of $\hat{\theta }$		x

### 6.2 Interval estimators

Straightforward use of the cumulative distribution function $F\left(\cdot |\tau^{2}\right)$ also yields a $100\left(1-\alpha \right)\%$ confidence interval for $\tau^{2}$:

$$\left\{\tau^{2}\geq 0:F\left(Q|\tau^{2}\right)\in \left[\alpha /2,1-\alpha /2\right]\right\}.$$

We use both the conditional estimated variances and the unconditional estimated variances in the Farebrother approximation to $Q_{F}$ (Section 5); we refer to the resulting profile estimators as FPC and FPU (“Farebrother Profile (Un)Conditional”) intervals. Jackson^22^ introduced a similar approach using conditional variances. The FPC interval can be obtained from the *confint* procedure in *metafor* ^7^ for “GENQ” or “GENQM” objects that used nbar weights. For the FPU intervals, we further distinguish between a “model‐based” version and a “naïve” version, according to whether we use ${\bar{p}}_{\mathrm{iT}}$ or ${\hat{p}}_{\mathrm{iT}}$ in $M_{2i}$ (8). Kulinskaya and Hoaglin^13^ give the higher unconditional moments of $\hat{\theta }$ required for the FPU intervals.

Our simulations (Section 7) also include the profile‐likelihood interval,^21^ the Q‐profile interval,^20^ and the KD interval.^10^ Table 1 gives the full list, and Section S1 in the Supporting Information gives further details.

## 7 SIMULATION DESIGN

Our simulation design followed that described in Bakbergenuly et al.^4^ Briefly, we varied five parameters: the overall true effect (θ), the between‐studies variance ($\tau^{2}$), the number of studies $\left(K\right)$, the studies' total sample size (n or $\bar{n}$, the average sample size), and the probability in the control arm ($p_{\mathrm{iC}}$). We kept the proportion of observations in the control arm (f) at 1/2.

The values of θ (0, 0.1, 0.5, 1, 1.5, and 2) aim to represent the range containing most values encountered in practice. LOR is a symmetric effect measure, so the sign of θ is not relevant.

The values of $\tau^{2}$ (0(0.1)1) systematically cover a reasonable range.

The numbers of studies (K=5, 10, and 30) reflect the sizes of many meta‐analyses and have yielded valuable insights in previous work.

In practice, many studies' total sample sizes fall in the ranges covered by our choices (n=20, 40, 100, and 250 when all studies have the same n, and $\bar{n}=30$, 60, 100, and 160 when sample sizes vary among studies). The choices of sample sizes corresponding to $\bar{n}$ follow a suggestion of Sánchez‐Meca and Marín‐Martínez,^23^ who constructed the studies' sample sizes to have skewness 1.464, which they regarded as typical in behavioral and health sciences. For K=5, Table 2 lists the sets of five sample sizes. The simulations for K=10 and K=30 used each set of unequal sample sizes twice and six times, respectively.

**TABLE 2:** Values of parameters in the simulations.

Parameter	Equal study sizes	Unequal study sizes
K (number of studies)	5, 10, 30	
n or $\bar{n}$ (average (individual) study size—total of the two arms) For K=10 and K=30, the same set of unequal study sizes is used twice or six times, respectively.	20, 40, 100, 250	30 (12,16,18,20,84), 60 (24,32,36,40,168), 100 (64,72,76,80,208), 160 (124,132,136,140,268)
f (proportion of observations in the control arm)	1/2	
$p_{\mathrm{iC}}$ (probability in the control arm)	0.1, 0.2, 0.5	
θ (true value of LOR)	0, 0.1, 0.5, 1, 1.5, 2	
$\tau^{2}$ (variance of random effects)	0 (0.1)1	

The values of $p_{\mathrm{iC}}$, 0.1, 0.2, and 0.5, provide a typical range of small to medium risks.

The values of $p_{\mathrm{iC}}$ and the true effect $\theta_{i}$ defined the probabilities $p_{\mathrm{iT}}$, and the counts $X_{\mathrm{iC}}$ and $X_{\mathrm{iT}}$ were generated from the respective binomial distributions. We used a total of 10,000 repetitions for each combination of parameters (which we also call a situation). We discarded “double‐zero” and “double‐*n*” studies and reduced the observed value of K accordingly. Next, we discarded repetitions with K<3 and used the observed number of repetitions for analysis.

The simulations used R statistical software.^24^ We used *metafor* for all methods of interest that it implemented. Table 1 gives the full list of point and interval estimators of $\tau^{2}$. The user‐friendly R programs implementing our methods are available on OSF.^25^

## 8 SIMULATION RESULTS

Our eprint^26^ reports the full simulation results. Here we describe the most important findings. We also refer to Figures S1 through S10 in the Supporting Information.

### 8.1 Bias in point estimation of $\tau^{2}$ for LOR

**FIGURE 1:**
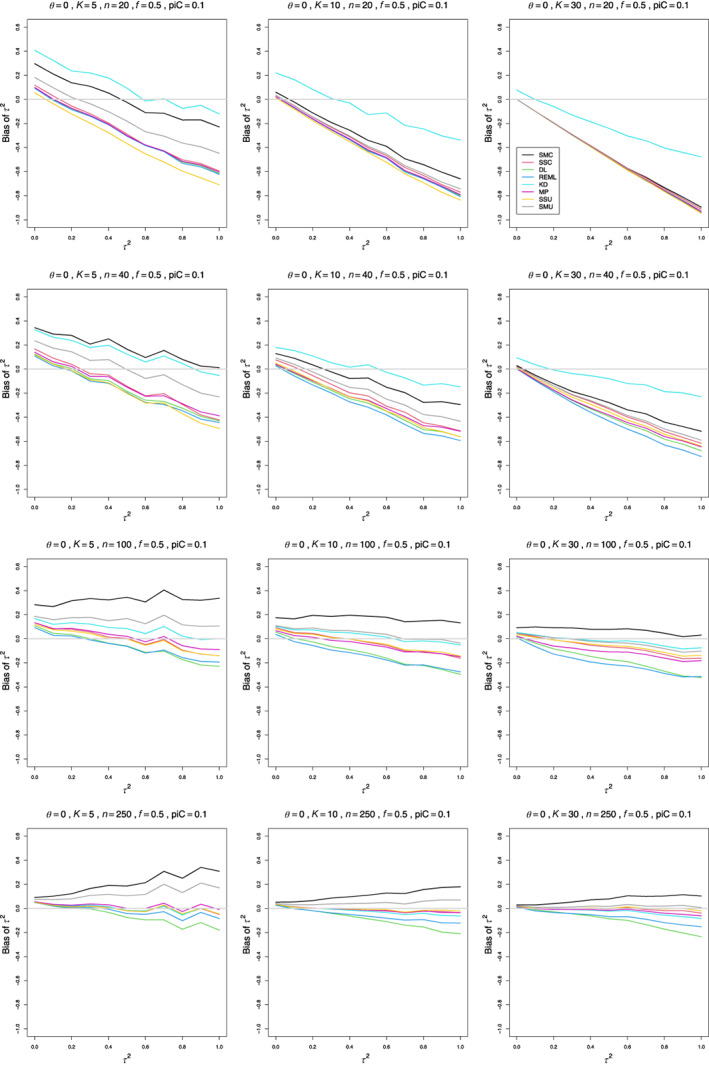
Bias of estimators of between‐study variance of LOR (the “only” versions of DL, REML, MP, and SMC; SSC “always”; KD; and the model versions of SMU and SSU) versus $\tau^{2}$, for equal sample sizes n=20,40,100, and 250, $p_{\mathrm{iC}}=0.1$, θ=0, and f=0.5. [Colour figure can be viewed at wileyonlinelibrary.com]

The relation of each estimator's bias to $\tau^{2}$ is roughly linear, with variation in intercept and, especially, in slope among the situations in the simulation. The slope varies most with n and K and to a lesser extent with $p_{\mathrm{iC}}$, θ, and whether sample sizes are equal or unequal. In one of the more extreme examples, when $p_{\mathrm{iC}}=.1$, θ=0, and K=5, the bias of SMC “only” when n=20 is +0.29 at $\tau^{2}=0$ and −0.22 at $\tau^{2}=1$, whereas when n=250, it is +0.09 at $\tau^{2}=0$ and +0.35 at $\tau^{2}=1$. The estimators' traces combine to form various patterns (Figure 1).

For small sample sizes, all estimators of $\tau^{2}$ have considerable bias, positive at $\tau^{2}=0$ and negative for larger $\tau^{2}$. The traces are roughly parallel. The pattern becomes tighter as K increases. When $p_{\mathrm{iC}}=0.1$, a few estimators have bias that is almost constant in $\tau^{2}$ when n≥100. When n=250, the traces form a fan‐shaped pattern, in which the bias ranges from 0.02 to 0.07 when $\tau^{2}=0$ and from −0.2 to +0.35 when $\tau^{2}=1$. The flattening and fan‐shaped pattern occur at somewhat smaller n as θ increases. The fan‐shaped pattern appears by n=100 when $p_{\mathrm{iC}}=0.2$ and by n=40 when $p_{\mathrm{iC}}=.5$. For larger sample sizes, some estimators have negative trends (or no trend), and other have positive trends.

The best estimators, almost unbiased when n≥250 and $p_{\mathrm{iC}}=0.1$, are MP “only,” KD, SSU model, and SSC “always.” The same estimators are recommended for larger values of $p_{\mathrm{iC}}$, where they generally become less biased earlier. When $p_{\mathrm{iC}}=0.2$ or $p_{\mathrm{iC}}=0.5$, various transitions occur at smaller sample sizes (Figures S1 and S2).

For comparison, our simulations included the popular point estimators DL and REML. We focus on their “only” version. The trace for the bias of DL “only” is in the middle of the traces for the collection of estimators, or slightly lower. When n=250 or $\bar{n}=160$, its negative bias, increasing in size as $\tau^{2}$ increases, stands out (usually alone).

The trace for REML “only” lies very close to that for DL, except that when n=250 (or, less often, $\bar{n}=160$), it is close to zero instead of trending negative.

To summarize, we recommend MP “only,” KD, SSU model, and SSC “always”; and we emphatically recommend against using DL and REML.

### 8.2 Median bias of estimators of $\tau^{2}$ for LOR

We define median bias as $P\left({\hat{\tau }}^{2}\geq \tau^{2}\right)-P\left({\hat{\tau }}^{2}\leq \tau^{2}\right)$. For a median, the median bias is zero.

**FIGURE 2:**
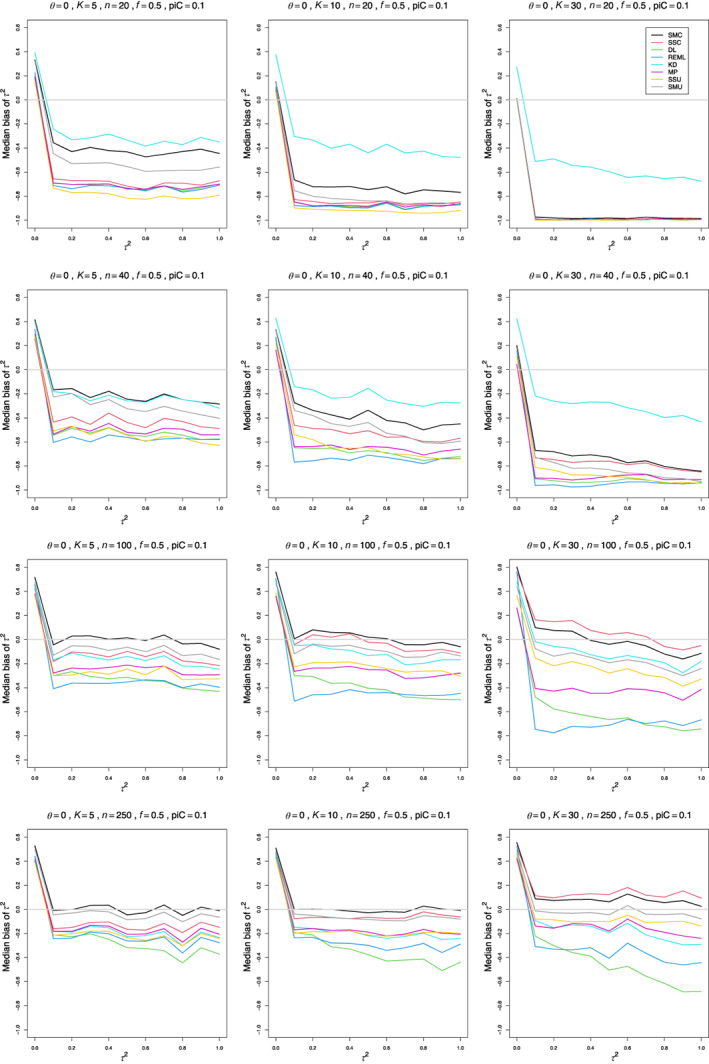
Median bias of estimators of between‐study variance of LOR (the “only” versions of DL, REML, MP, and SSC; SMC “always”; KD; and the model versions of SMU and SSU) versus $\tau^{2},$ for equal sample sizes n=20,40,100 and 250, $p_{\mathrm{iC}}=0.1$, θ=0 and f=0.5. [Colour figure can be viewed at wileyonlinelibrary.com]

For $0.1\leq \tau^{2}\leq 1$, all estimators have negative bias for small to medium sample sizes, n≤40 (Figure 2). Interestingly, the median bias of most estimators is almost constant across the range of nonzero $\tau^{2}$ values. That level, however, varies with n and K. As n increases, the bias becomes less negative (but not small), but increasing K tends to make it more negative. DL “only,” REML “only,” and MP “only” depart from the almost constant pattern. As K increases, the trace of DL “only” declines steeply (e.g., from −0.20 at $\tau^{2}=0.1$ to −0.68 at $\tau^{2}=1$ when $p_{\mathrm{iC}}=0.2$, θ=0, n=100, and K=30, Figure S3), and the trace of REML “only” declines less steeply (and only when $p_{\mathrm{iC}}=0.5$, Figure S4). The trace of MP “only” tends to have a positive slope when n=40 and n=100 and θ≥1 and $p_{\mathrm{iC}}=0.1$ and a negative slope when n=20 and n=40 and $p_{\mathrm{iC}}=0.5$.

When n=20 or $\bar{n}=30$ and $p_{\mathrm{iC}}=0.1$, none of the estimators are satisfactory. KD comes closest, with median bias around −0.25 at best. For $p_{\mathrm{iC}}=0.2$ and $p_{\mathrm{iC}}=0.5$, SMC “always” and SMC “only” are better; in a few situations one or both have small positive or negative bias.

The majority of the standard estimators have negative bias for larger values of n. This means that their median values of ${\hat{\tau }}^{2}$ are too low. When n≥100 for $p_{\mathrm{iC}}=0.1$ and n≥40 for $p_{\mathrm{iC}}\geq 0.2$, the new median‐unbiased estimators perform well. We recommend SMC “always” and SMU model. Both consistently result in almost median‐unbiased estimation across the range of $p_{\mathrm{iC}}$ values.

### 8.3 Coverage of interval estimators of $\tau^{2}$ for LOR

The results of our simulations show a few general patterns, but specific choices of interval estimators depend on values of parameters or combinations of parameters. In particular, we often separate K=30 from K=5 and K=10.

At $\tau^{2}=0$ coverage is always too high (essentially 1.00). For small n, it is roughly flat when $\tau^{2}\geq 0.1$ and K=5 or K=10.

**FIGURE 3:**
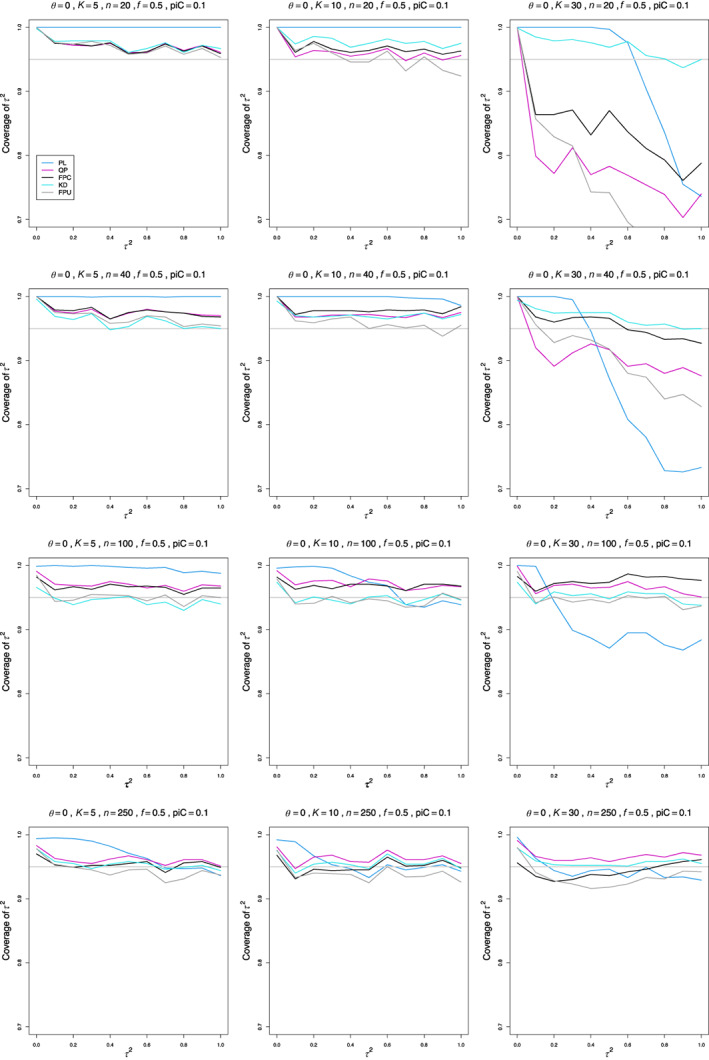
Coverage of 95% confidence intervals for between‐study variance of LOR (the “only” versions of PL, QP and FPC; KD; and the model version of FPU) versus $\tau^{2}$, for equal sample sizes n=20,40,100 and 250, $p_{\mathrm{iC}}=0.1$, θ=0_,_ and f=0.5. [Colour figure can be viewed at wileyonlinelibrary.com]

When $p_{\mathrm{iC}}=0.1$ and θ=0, coverage of most estimators remains too high when n≤100 (Figure 3). KD and FPU model are close to 0.95 when n=100. When K=30 and n=20 or n=40, all estimators except KD break down: most have coverage <0.90 at $\tau^{2}=0.1$, and their coverage declines steeply as $\tau^{2}$ increases. Some form of breakdown persists for all θ. As θ increases, and n=20 or n=40, coverage at $0.1\leq \tau^{2}\leq 1$ moves toward 0.95. KD and FPC model (for n≥40) seem the best choices.

When $p_{\mathrm{iC}}=0.2$ or $p_{\mathrm{iC}}=0.5$ (Figures S5 and S6), the picture is simpler. Coverage when n=20 or n=40 becomes closer to (or equals) 0.95 as $p_{\mathrm{iC}}$ increases, except for the “always” versions of PL, QP, and FPC when $p_{\mathrm{iC}}=0.2$. For K=30, KD seems the best single choice.

Coverage of PL is usually too high, and in some situations it seems trapped at 1.00.

### 8.4 Left and right coverage error

It is often informative to approach estimation of coverage by separating its complement, the coverage error or “miscoverage,” into two parts, corresponding to whether the value of the parameter is to the left of the lower confidence limit or to the right of the upper confidence limit. Efron and Tibshirani in Reference [27], section 13.5 denote these by “miss left” and “miss right.” A CI that had a small miss‐left percentage would overcover on the left; if it had a large percentage, it would undercover. The confidence intervals that we studied aim to have miscoverage equal to 2.5% on each side (Section 6.2), but an approximation for the distribution of Q may not provide the desired balance, even if the overall coverage is close to 95%. Thus, our simulations included the miss‐left and miss‐right percentages.

**FIGURE 4:**
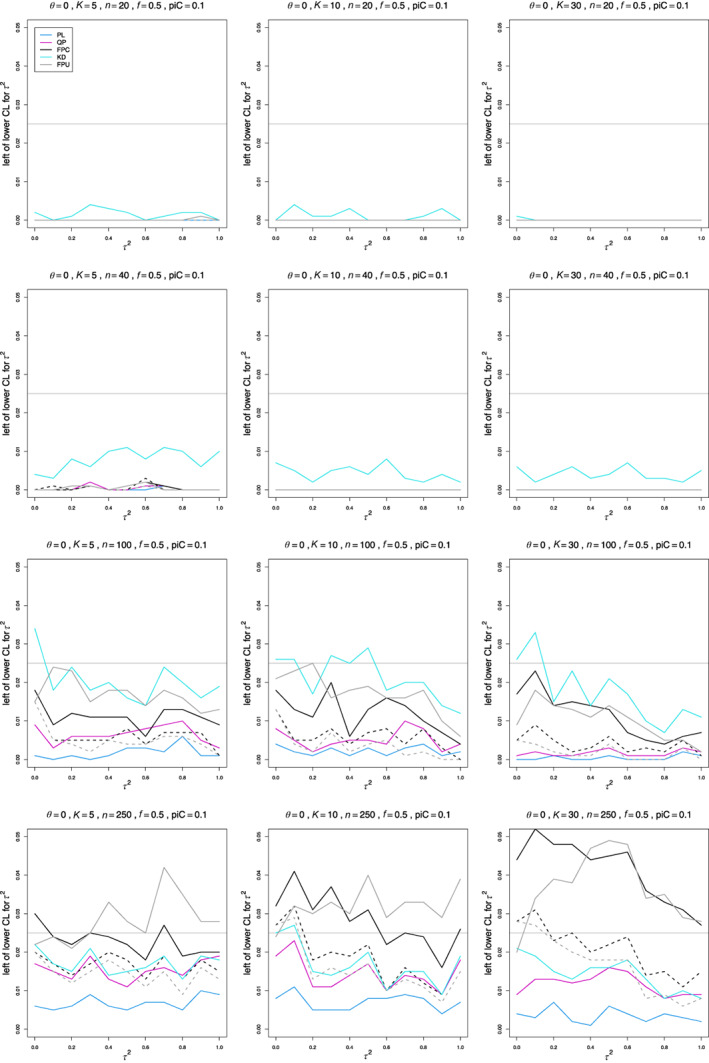
Miss‐left probability of PL, QP, KD, FPC, and FPU 95% confidence intervals for between‐study variance of LOR versus $\tau^{2}$, for equal sample sizes n=20,40,100 and 250, $p_{\mathrm{iC}}=0.1$, θ=0, and f=0.5. Solid lines: the “only” versions of PL, QP and FPC; KD; and the model version of FPU. Dashed lines: the “always” version of FPC and the naïve version of FPU. [Colour figure can be viewed at wileyonlinelibrary.com]

In general, the miss‐left percentages are typically lower than 2.5% for small sample sizes and/or control‐arm probabilities, but they improve for n≥100 and for larger $p_{\mathrm{iC}}$ (Figures 4, S7, S8). A low miss‐left percentage means that the confidence interval includes an excess of low $\tau^{2}$ values.

The miss‐left percentages of KD, FPU naïve, and FPC “always” are closer to 2.5% from n=100, and miss‐left percentages of QP are typically lower than nominal when $p_{\mathrm{iC}}=0.1$ but improve for larger $p_{\mathrm{iC}}$.

The only exceptions to typically lower than nominal miss‐left percentages are the FPC “only” and FPU model intervals, whose percentages for n≥40 are often higher than nominal for K=30 and sometimes when K=10. KD occasionally has high percentages for high values of θ when K=30. PL miss‐left percentages are especially low for all sample sizes.

**FIGURE 5:**
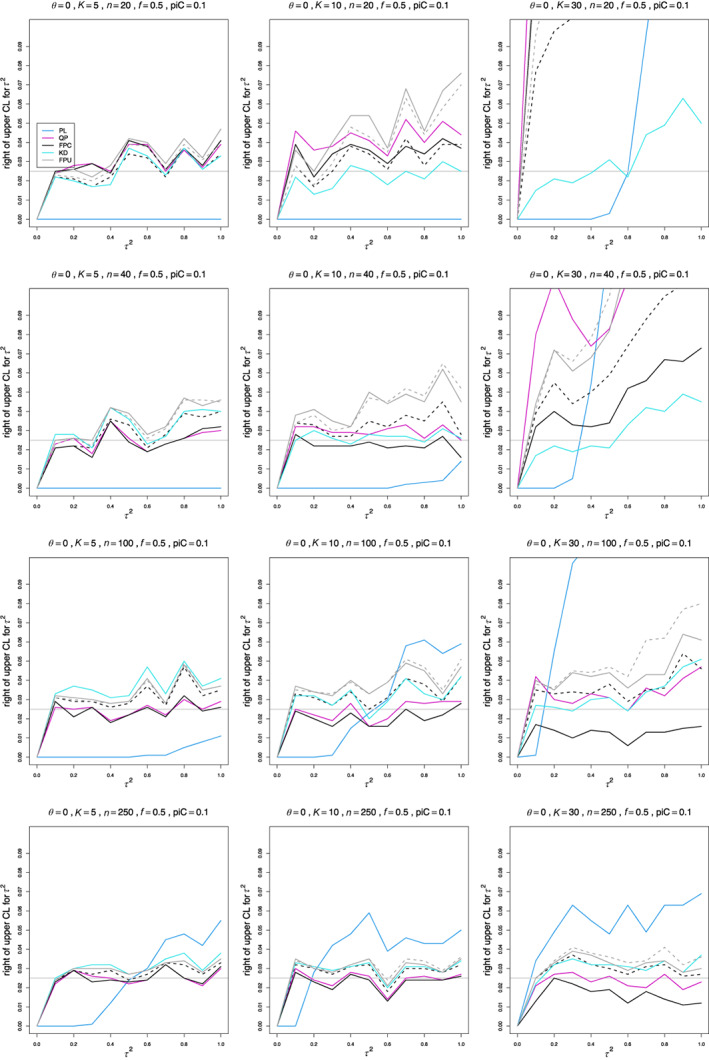
Miss‐right probability of PL, QP, KD, FPC, and FPU 95% confidence intervals for between‐study variance of LOR versus $\tau^{2}$, for equal sample sizes n=20,40,100 and 250, $p_{\mathrm{iC}}=0.1$, θ=0, and f=0.5. Solid lines: the “only” versions of PL, QP and FPC; KD; and the model version of FPU. Dashed lines: the “always” version of FPC and the naïve version of FPU. [Colour figure can be viewed at wileyonlinelibrary.com]

The miss‐right percentages are often higher than nominal, but they improve for larger n and $p_{\mathrm{iC}}$ (Figures 5, S9, S10). K=30 is especially challenging.

For $p_{\mathrm{iC}}=0.1$, the miss‐right percentages of KD, QP “always,” and FPC “always” are closest to 2.5% when n=20 and K=5, but increase for larger n. When K=5 or 10, the percentages of FPC “only” and QP “only” are reasonably close to 2.5% when n≥40. The percentages of KD are consistently close to 2.5% for K=10. For K=30, the range of results is larger. The percentages of KD, QP “only,” and FPC “always” are close to 2.5% when n≥100. The percentages for all other estimators are typically higher, with the exception of FPU model, which has lower than nominal percentages. PL produces erratic percentages, from 0 to above 10%. For larger values of $p_{\mathrm{iC}}$, the miss‐right percentages of all estimators, with the exception of PL, improve earlier, so that those of KD, QP “only,” and FPC “always” are all close to 2.5% when n=40 and $p_{\mathrm{iC}}=0.5$, even when K=30.

Lack of balance in the miss‐left and miss‐right percentages for small sample sizes and/or control‐arm probabilities agrees with our findings^13^ that the chi‐square approximation for $Q_{\mathrm{IV}}$ and the Farebrother approximation for $Q_{F}$ are accurate only for larger sample sizes.

## 9 EXAMPLE: SMOKING CESSATION

Stead et al.^28^ conducted a systematic review of clinical trials on the use of physician advice for smoking cessation. We use the data from the subgroup of interventions in which the treatment involved only one visit (Comparison 3.1.4, p. 54). The first version of the report was published in 2001. In an update, published in 2004, 17 studies included this comparison. The 2013 update included one more study, by Unrod (2007). For each study, Table S1 gives the number of subjects in the treatment and control arms and the number who were nonsmokers at the longest follow‐up time reported (either 6 months or 12 months). The definition of “nonsmoker” varied among the studies. Some studies required sustained abstinence, and others only asked about smoking status at that time. Stead et al.^28^ analyzed relative risk. Kulinskaya and Hoaglin^6^ showed that both OR and RR are reasonable effect measures for these data. Here we consider estimation of $\tau^{2}$ in two meta‐analyses of LOR.

The studies were mostly balanced, though two studies had substantially more subjects in the treatment arm. Sample sizes varied from 182 to 3128, with an average of 836 patients per study. The mean probabilities of smoking cessation in both arms were rather low, at 0.058 in the treatment arm and 0.043 in the control arm.

The standard IV‐based meta‐analysis of LOR for the original 17 studies gives $\hat{\theta }=0.4774$ with standard error 0.1148 and p<0.0001 for the intervention effect (${\hat{\tau }}_{\mathrm{MP}}^{2}=0.0754$). The fixed‐weights estimate of θ is higher, at 0.7127. In testing for heterogeneity, $Q_{\mathrm{IV}}=24.84$, and the chi‐square approximation on 16 df provides a p‐value of .079 ($I^{2}=38.18\%$). The *p*‐values for the KD and F SSW naïve methods (which Kulinskaya and Hoaglin^6^ recommend) are very close, at 0.035 and 0.038, respectively.

Table 3 shows striking differences: the SSC and SSU estimates of $\tau^{2}$ are more than twice as large as the standard IV estimates. This agrees with our simulations, which showed large positive biases of SSC and SSU for low values of $\tau^{2}$. All confidence intervals have zero as the lower limit (Table 4). This result contradicts the significant *p*‐values from the KD and F SSW naïve tests, but it can be explained by the inflated confidence level of all six confidence intervals near zero. Adding 1/2 to the numbers of events somewhat reduces the estimated values and the upper confidence limits. This would somewhat reduce the positive biases and the inflation in coverage. Because of the large sample sizes, there are only minor differences between conditional and unconditional model‐based or naïve estimators of $\tau^{2}$ and confidence intervals.

**TABLE 3:** Estimated values of $\tau^{2}$ in the meta‐analysis of LOR for the data of Stead et al. on the use of physician advice for smoking cessation.

Example	Add 1/2	DL	REML	MP	KD	SSC	SSU model	SSU naïve	SMC	SMU model	SMU naïve
Stead et al. (17 studies)	Always	0.0627	0.0696	0.0701	0.0887	0.1715	0.1725	0.1680	0.2014	0.2043	0.1972
	Only	0.0675	0.0769	0.0754		0.1776			0.2098		
Stead et al. (18 studies)	Always	0.0514	0.0537	0.0576	0.0740	0.1634	0.1642	0.1601	0.1913	0.1939	0.1873
	Only	0.0555	0.0597	0.0623		0.1697			0.1997		

**TABLE 4:** 95% confidence intervals for the between‐study variance in the meta‐analysis of LOR for the data of Stead et al. on the use of physician advice for smoking cessation.

Example	Add 1/2	QP	PL	KD	FPC	FPU model	FPU naïve
Stead et al. (17 studies)	Always	[0, 0.3745]	[0, 0.3445]	[0, 0.3910]	[0, 0.7583]	[0, 0.7554]	[0, 0.7389]
	Only	[0, 0.3957]	[0, 0.3713]		[0, 0.8075]		
Stead et al. (18 studies)	Always	[0, 0.3267]	[0, 0.2915]	[0, 0.3419]	[0, 0.6992]	[0, 0.6969]	[0, 0.6820]
	Only	[0, 0.3455]	[0, 0.3139]		[0, 0.7458]		

Addition of the 18th study somewhat increased the *p*‐value for the standard Q test, to 0.107, and the KD *p*‐value to 0.052, but hardly affected the *p*‐values of the SSW‐based methods. The recommended F SSW naïve test rejects homogeneity at the 5% significance level, with p=0.033. The estimated $\tau^{2}$ values are somewhat smaller, and all confidence intervals still start at zero.

**FIGURE 6:**
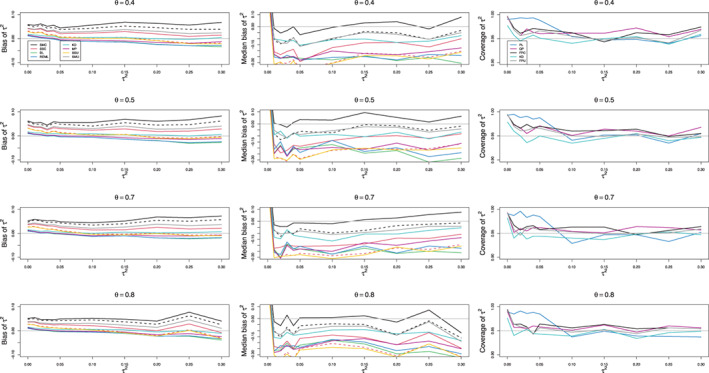
Bias, median bias, and coverage at 95% nominal confidence level of point and interval estimators of $\tau^{2}$ for the example of meta‐analysis by Stead et al. Solid lines: the “only” versions of DL, REML, MP, SSC, and SMC; KD; the model versions of SSU and SMU point estimators; the “only” versions of PL, QP, and FPC; FPU model, and KD intervals. Dashed lines: the “always” versions of SSC and SMC point estimators. [Colour figure can be viewed at wileyonlinelibrary.com]

To better understand the properties of the estimation methods for very small values of $p_{\mathrm{iC}}$ in this example, we performed additional simulations with 1000 repetitions in the relevant intervals of values, $\tau^{2}\in \left[0,\;0.30\right]$ and $\theta \in \left[0.4,\;0.8\right]$, keeping the sample sizes as in the example and using the probabilities $p_{\mathrm{iC}}=X_{\mathrm{iC}}/n_{\mathrm{iC}}$. Figure 6 shows the results. In agreement with our main series of simulations, KD, MP “only”, SSC “always,” and SSU model are the least biased estimators of $\tau^{2}$, whereas SMC “only” is the least median‐biased. Table S2 gives summary statistics for these five estimators when $\tau^{2}=0.05$ and 0.2, and θ=0.5 and 0.7. Additionally, Table S2 provides summary statistics when θ=0.5 and $\tau^{2}=0.2$, but the control arm probabilities are less extreme, at $p_{\mathrm{iC}}=\left(X_{\mathrm{iC}}/n_{\mathrm{iC}}\right)+.1$.

**FIGURE 7:**
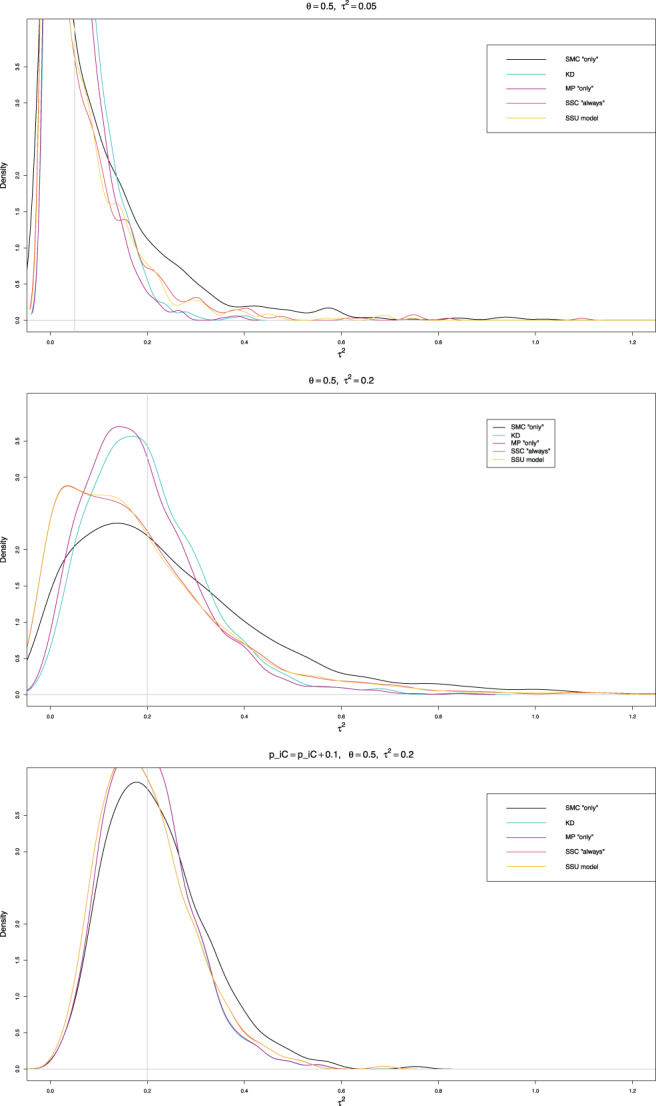
Density plots of the point estimators of $\tau^{2}$ for the example of meta‐analysis by Stead et al.: the “only” versions of MP and SMC; the “always” version of SSC; the model version of SMU; and KD. [Colour figure can be viewed at wileyonlinelibrary.com]

Both lower and upper quartiles for the first four estimators of $\tau^{2}$ are comparable, though the upper quartile of SMC “only” is somewhat higher. However, the three effective‐sample‐size estimators have a much longer right tail than KD and MP “only.” This shape explains their much higher mean values. Comparing the medians, SMC “only” is almost median‐unbiased in all five scenarios, whereas SSC “always” and SSU model have the lowest medians, followed by MP “only” and then KD. This pattern is especially noticeable when the control arm probabilities are low. The lower the median, the more often these estimators would underestimate true heterogeneity. The differences between distributions decrease quite considerably for larger control arm probabilities, so the KD and MP “only” densities practically coincide, as do the densities of SSC “always” and SSU model (Figure 7). Overall, KD is the best mean‐unbiased estimator, and SMC “only” is the best median‐unbiased estimator. Which one to prefer depends very much on the context of the research. All confidence intervals provide reasonable coverage, though KD is sometimes too liberal, and PL too conservative (Figure 6).

## 10 DISCUSSION

Estimation of heterogeneity variance $\tau^{2}$ is an important part of any meta‐analysis. Apart from its importance per se, it also affects the value of an estimated pooled effect and its estimated variability. In this paper, we studied estimation of $\tau^{2}$ based on $Q_{F}$, a generalized Q statistic with fixed effective‐sample‐size weights, and compared four proposed new point estimators of $\tau^{2}$ (SSC, SMC, SSU, and SMU) and two new interval estimators (FPC and FPU) with traditional estimators based on the Q statistic with inverse‐variance weights, $Q_{\mathrm{IV}}$.

The new point estimators based on the expected value of $Q_{F}$ involve estimates (${\hat{v}}_{i}$) of the variances of the ${\hat{\theta }}_{i}$. We obtained SSC from the conditional variances and SSU from the unconditional variances of the ${\hat{\theta }}_{i}$. Similarly, our novel point estimators based on the median of the Farebrother approximation to the distribution of $Q_{F}$ are SMC and SMU, and our profile interval estimators are FPC and FPU.

For each unconditional estimator, we investigated two approaches to estimation of $p_{\mathrm{iT}}$ (which is used in the calculation of the second and fourth central moments of ${\hat{\theta }}_{i}$), “naïve” estimation of $p_{\mathrm{iT}}$ from $X_{\mathrm{iT}}$ and $n_{\mathrm{iT}}$ and “model‐based” estimation using the fixed‐effects meta‐analysis estimate of the overall effect to obtain ${\bar{p}}_{\mathrm{iT}}$ from ${\hat{p}}_{\mathrm{iC}}$. Additionally, we considered two versions of the traditional and the unconditional estimators of $\tau^{2}$: adding 1/2 to the four cell counts always, or only when one of those counts is zero. Thus, our simulation study examined a total of 15 point estimators of $\tau^{2}$ and 9 interval estimators (Table 1).

For mean bias, the best estimators are MP “only,” KD, SSU model, and SSC “always.” The DL and REML estimators have the worst bias. For median bias, which was not studied previously, an important finding is that the majority of the standard estimators have negative bias for larger values of n. This means that their median values of ${\hat{\tau }}^{2}$ are too low. We recommend SMC “always” and SMU model. Both consistently result in almost median‐unbiased estimation across the range of $p_{\mathrm{iC}}$ values for moderate to large n.

Coverage of the confidence intervals depends on $p_{\mathrm{iC}}$, n, and K. There is also some dependence on θ and $\tau^{2}$. However, KD, FPC “always,” and FPU model are the best choices overall. PL is the worst choice. We also considered left and right coverage errors separately. Typically, the miss‐left rates are below, and the miss‐right rates are above, the nominal 2.5% level, especially so for small sample sizes and/or control arm probabilities. Arguably, this is not bad, as it would reduce the width of the confidence intervals.^29^ Lack of balance in the miss‐left and miss‐right rates agrees with our findings^13^ that the chi‐square approximation for $Q_{\mathrm{IV}}$ and the Farebrother approximation for $Q_{F}$ are accurate only for larger sample sizes.

Alongside the novel two‐stage estimators using sample‐size‐based weights, our simulation study involved a variety of established inverse‐variance‐based two‐stage estimators of heterogeneity. With a very few exceptions, such as MP and, especially, KD, their performance is not impressive. One‐stage meta‐analysis is often suggested as a better choice. However, we previously considered quality of one‐stage estimation in GLMM‐based binomial‐normal models.^11^, ^30^ and found it lacking. Also, it is useful to remember that GLMMs are still asymptotic methods that use normal likelihood.

Ideally, for meta‐analyses of log‐odds‐ratio, a single point estimator of $\tau^{2}$ would have acceptably low bias (and, perhaps, median bias) for a substantial region of $p_{\mathrm{iC}}$, θ, n, K, and $\tau^{2}$. Similarly, a single confidence interval would have close to nominal coverage of $\tau^{2}$. Our results show some progress toward those goals, by demonstrating advantages of new estimators in some situations and, importantly, by demonstrating that some popular estimators have unacceptable bias or coverage. The goals, however, require further research. In the interim, effective education can help users avoid methods that perform poorly.

## AUTHOR CONTRIBUTIONS

**Elena Kulinskaya:** Conceptualization; funding acquisition; methodology; software; visualization; writing – original draft; writing – review and editing. **David C. Hoaglin:** Conceptualization; investigation; methodology; writing – original draft; writing – review and editing.

## CONFLICT OF INTEREST STATEMENT

The authors declare no conflicts of interest.

## Supporting information


**Data S1:** Supporting Information.

## Data Availability

Our full simulation results are available as an arXiv e‐print (arXiv:2208.00707v1). The user‐friendly R program implementing all studied estimators of heterogeneity variance \$\tau^2\$ in meta‐analysis of log‐odds‐ratio with related confidence intervals is available at https://osf.io/5n3vd
