# Supplementary material for: Estimation of heterogeneity variance based on a generalized Q statistic in meta‐analysis of log‐odds‐ratio
Source: Res Synth Methods. 2023 Jun 28;14(5):671–88. doi: 10.1002/jrsm.1647 (PMC10946484; doi:10.1002/jrsm.1647)
Supplement: Supplementary file 1 — Data S1: Supporting Information. [file JRSM-14-671-s001.pdf]

Supporting information to Estimation of heterogeneity variance based on a generalized Q statistic in meta-analysis of log-odds-ratio

By Elena Kulinskaya and David C. Hoaglin

June 15, 2023

Appendices

S1 Confidence intervals for τ^2 based on Q_{IV}

S1.1 Profile-likelihood confidence interval

Hardy and Thompson [1996] base the profile-likelihood (PL) confidence interval on the normal-normal random-effects model, in which $\hat{\theta}_i \sim N(\theta_i, \sigma_i^2)$ and $\theta_i \sim N(\theta, \tau^2)$, and hence the marginal distribution for $\hat{\theta}_i$ is $N(\theta, \sigma_i^2 + \tau^2)$. As usual, only estimates of the σ_i^2 are available; but assuming that those estimates are the true values allows the normal likelihood to be a function of only θ and τ^2. They obtain the PL confidence interval for τ^2 by profiling the

log-likelihood, $l(\theta, \tau^2)$, as a function of τ^2; that is, for each τ_0^2, they obtain the maximum-likelihood estimate of θ, $\tilde{\theta}(\tau_0^2)$. The confidence interval comes from the likelihood-ratio test with the null hypothesis $H_0 : \tau^2 = \tau_0^2$, in which the test statistic is

$$\lambda(\hat{\theta}_1, \ldots, \hat{\theta}_K) = -2[l(\tilde{\theta}(\tau_0^2), \tau_0^2) - l(\hat{\theta}, \hat{\tau}^2)]$$

and $(\hat{\theta}, \hat{\tau}^2)$ is the overall maximum-likelihood estimate of (θ, τ^2). For random samples of $\hat{\theta}_1, \ldots, \hat{\theta}_K$, the distribution of $\lambda(\hat{\theta}_1, \ldots, \hat{\theta}_K)$ approaches the chi-square distribution on 1 degree of freedom as K becomes large. The 95% PL confidence interval consists of the values of τ^2 for which the test does not reject H_0. Thus, Hardy and Thompson define it by

$$l(\tilde{\theta}(\tau^2), \tau^2) > l(\hat{\theta}, \hat{\tau}^2) - 3.84/2.$$

This approach has several limitations. It does not account for sampling variation in the estimates of the σ_i^2. For the OR, the distribution of $\log(OR)$ in small samples is not well approximated by a normal distribution. The chi-square distribution on 1 d.f. is the asymptotic distribution of $\lambda(\hat{\theta}_1, \ldots, \hat{\theta}_K)$ as K becomes large. Also, as Viechtbauer [2007] pointed out, $\tau^2 = 0$ lies on the boundary of the parameter space; in that case, the asymptotic distribution is a 50–50 mixture of χ_1^2 and a degenerate distribution at 0.

S1.2 Q-profile confidence interval

The Q-profile (QP) confidence interval [Viechtbauer, 2007] uses $w_i = 1/(\sigma_i^2 + \tau^2)$, yielding $Q(\tau^2)$. In the normal-normal random-effects model (treating the σ_i^2 as known constants), Viechtbauer refers $Q(\tau^2)$ to the chi-square distribution on $K-1$ d.f., whose .025 and .975 quantiles are $\chi^2_{K-1,.025}$ and $\chi^2_{K-1,.975}$. Solving (iteratively)

$$Q(\tilde{\tau}_L^2) = \chi^2_{K-1,.975} \qquad \text{and} \qquad Q(\tilde{\tau}_U^2) = \chi^2_{K-1,.025}$$

yields $(\tilde{\tau}_L^2, \tilde{\tau}_U^2)$ as the 95% QP confidence interval.

In Viechtbauer's simulations, for LOR, coverage of the QP interval is substantially closer to .95 than that of the PL interval. The QP interval, however, faces the potential shortcoming that, with the $\hat{\sigma}_i^2$ instead of the σ_i^2 in the weights, $Q(\tau^2)$ does not follow a chi-square distribution, even when $\tau^2 = 0$ [Hoaglin, 2016].

S1.3 Kulinskaya-Dollinger confidence interval

Instead of assuming that $\sigma_i^2 = \hat{\sigma}_i^2$, Kulinskaya and Dollinger [2015] took into account the sampling variability in the $\hat{\sigma}_i^2$. Using theoretical results and simulations, they obtained a corrected formula that approximates the first moment of the null distribution of Q_{IV} for LOR and a formula (as a function of that corrected first moment) for the corresponding second moment. Both corrected moments are smaller than those of the usual chi-square distribution. Because the full details are complicated, we do not show the corrected moments here. Kulinskaya and Dollinger [2015] give some details and include a link to an R program for the calculations. The KD confidence interval is obtained by referring $Q(\tau^2)$ to a gamma distribution with the corrected two moments.

References

Rebecca J. Hardy and Simon G. Thompson. A likelihood approach to meta-analysis with random effects. *Statistics in Medicine*, 15:619–629, 1996.

David C. Hoaglin. Misunderstandings about Q and “Cochran’s Q test” in meta-analysis. *Statistics in Medicine*, 35:485–495, 2016.

Elena Kulinskaya and Michael B. Dollinger. An accurate test for homogeneity of odds ratios based on Cochran’s Q-statistic. *BMC Medical Research Methodology*, 15(1):49, 2015.

Wolfgang Viechtbauer. Confidence intervals for the amount of heterogeneity in meta-analysis. *Statistics in Medicine*, 26(1):37–52, 2007.

S2 Supplemental Figures

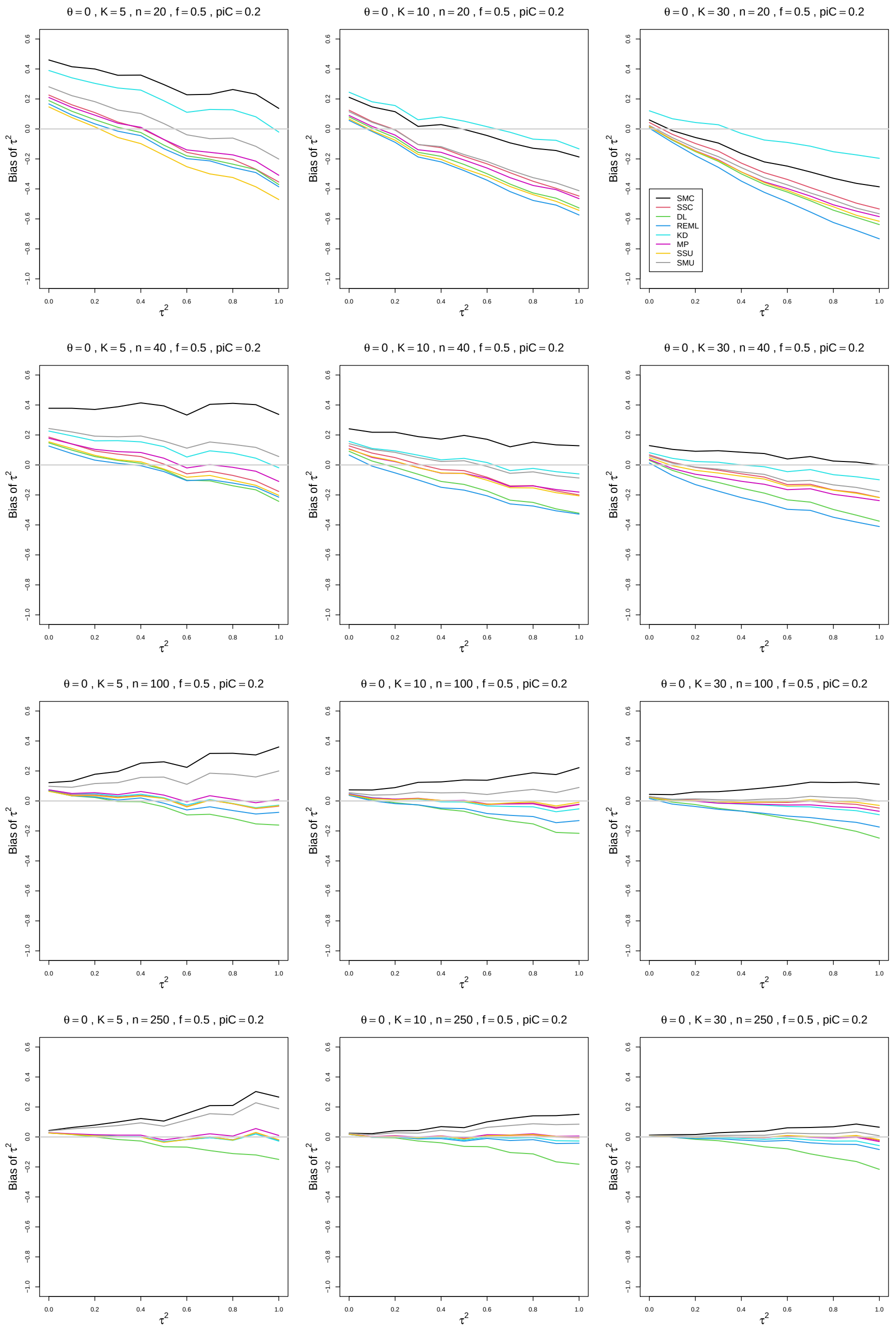

Figure S1: Bias of estimators of between-study variance of LOR (the "only" versions of DL, REML, MP, and SMC ; SSC "always"; KD; and the model versions of SMU and SSU) vs τ^2, for equal sample sizes $n = 20,\ 40,\ 100$ and 250, $p_{iC} = .2$, $\theta = 0$ and $f = 0.5$.

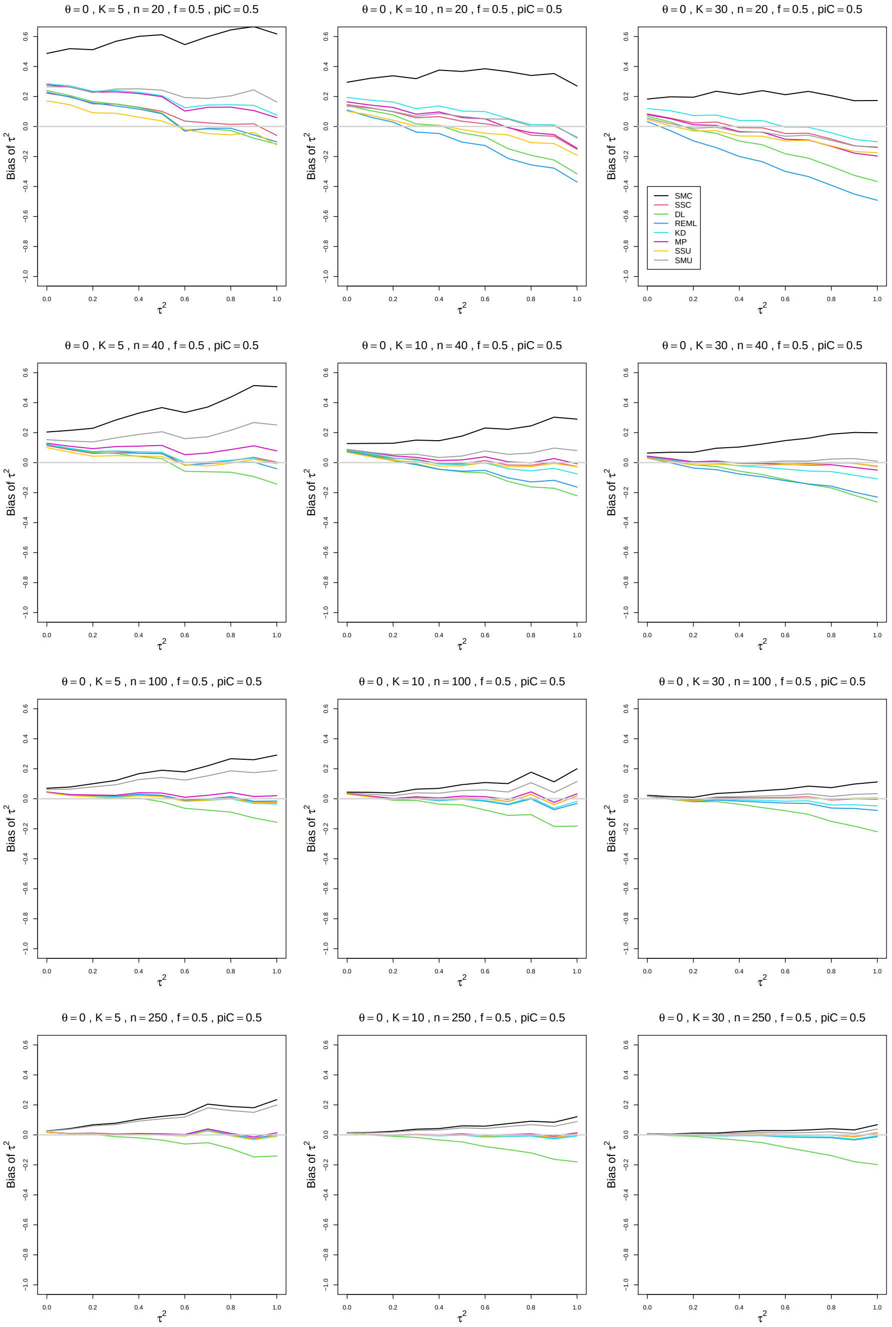

Figure S2: Bias of estimators of between-study variance of LOR (the "only" versions of DL, REML, MP, and SMC ; SSC "always"; KD; and the model versions of SMU and SSU) vs τ^2, for equal sample sizes $n = 20,\ 40,\ 100$ and 250, $p_{iC} = .5$, $\theta = 0$ and $f = 0.5$.

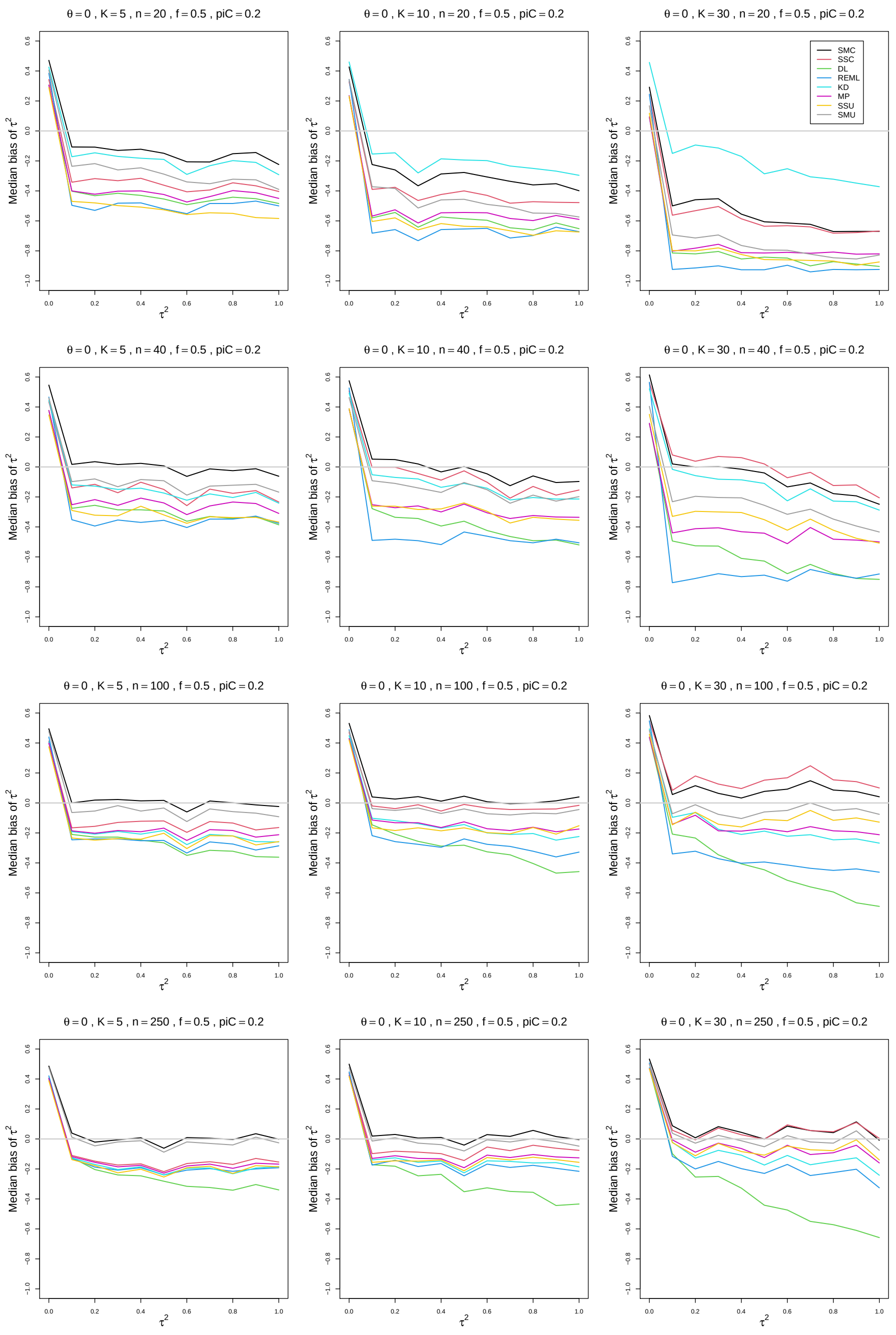

Figure S3: Median bias of estimators of between-study variance of LOR (the "only" versions of DL, REML, MP, and SSC ; SMC "always"; KD; and the model versions of SMU and SSU) vs τ^2, for equal sample sizes $n = 20,\ 40,\ 100$ and 250, $p_{iC} = .2$, $\theta = 0$ and $f = 0.5.8$

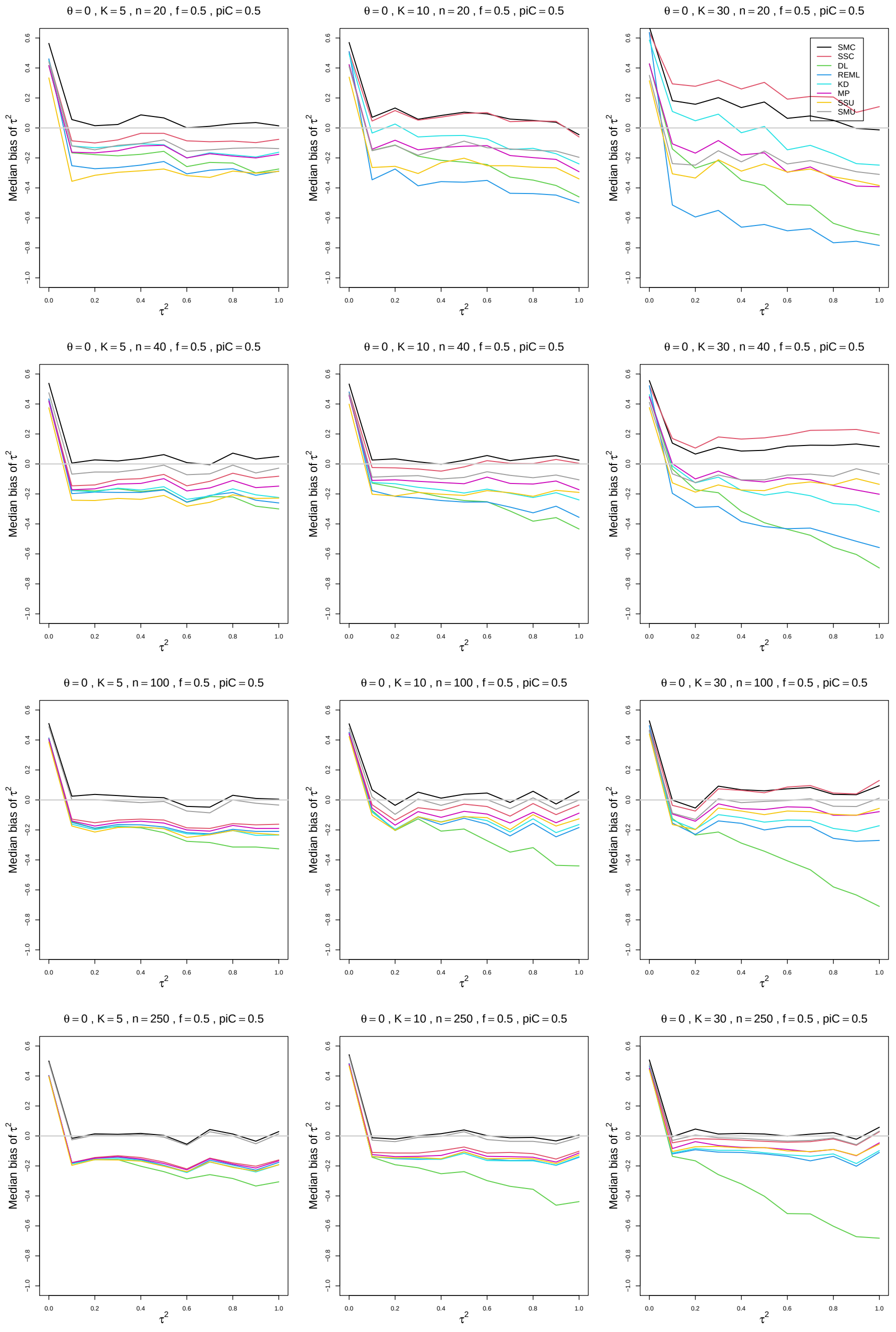

Figure S4: Median bias of estimators of between-study variance of LOR (the “only” versions of DL, REML, MP, and SSC ; SMC “always”; KD; and the model versions of SMU and SSU) vs τ^2, for equal sample sizes $n = 20,\ 40,\ 100$ and 250, $p_{iC} = .5$, $\theta = 0$ and $f = 0.5.9$

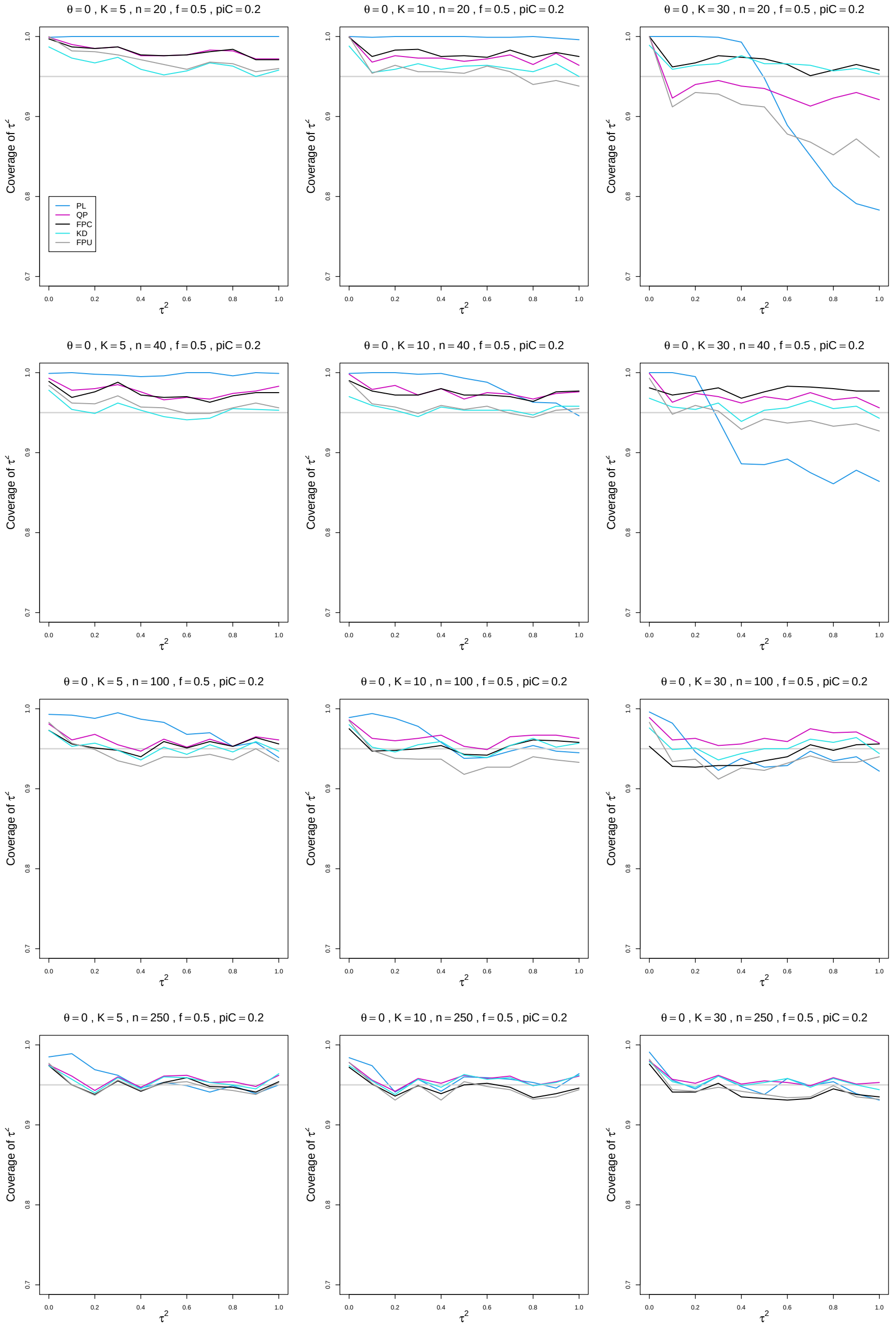

Figure S5: Coverage of 95% confidence intervals for between-study variance of LOR (the “only” versions of PL, QP and FPC; KD; and the model versions of FPU) vs τ^2, for equal sample sizes $n = 20,\ 40,\ 100$ and 250, $p_{iC} = .2$, $\theta = 0$ and $f = 0.5$.

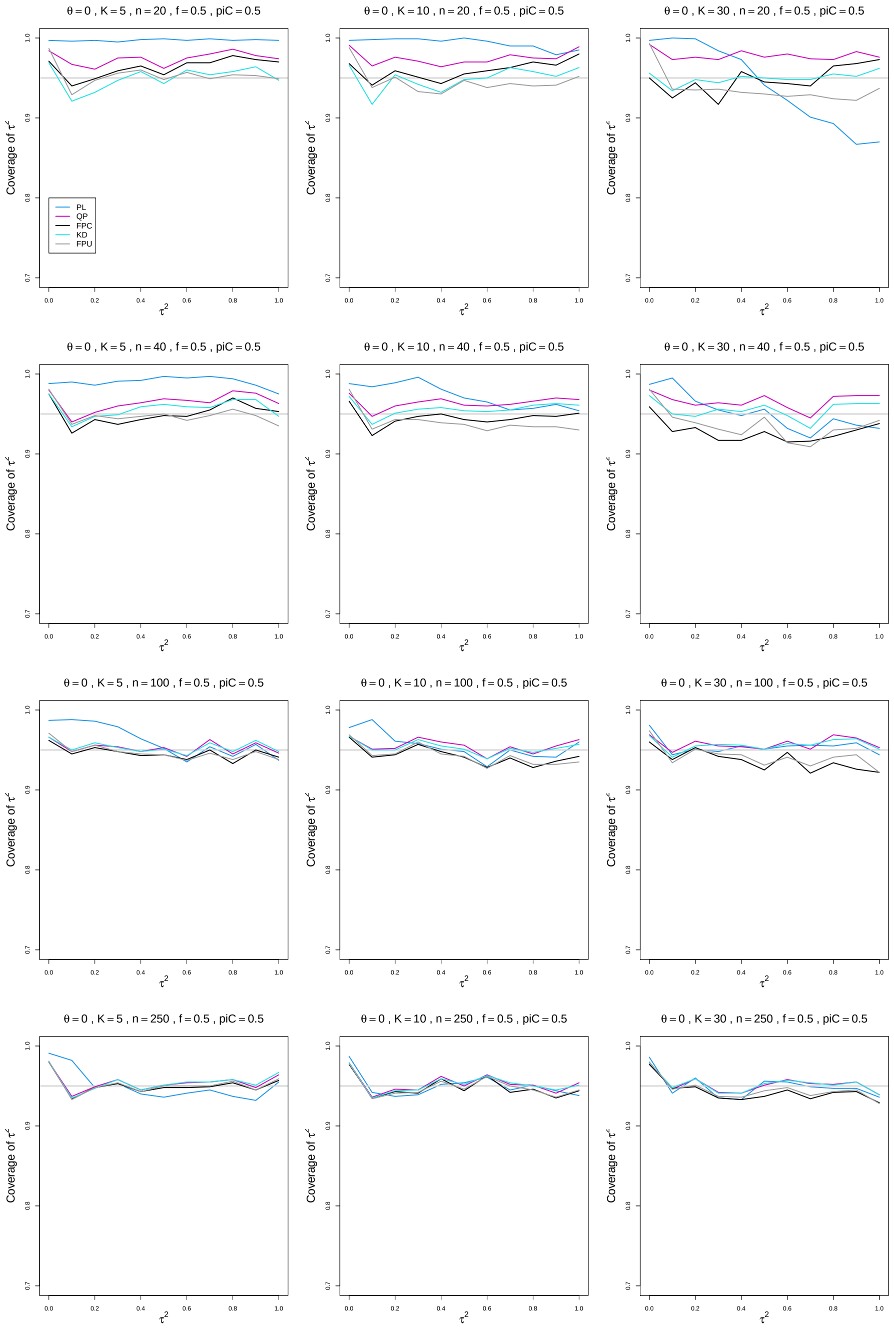

Figure S6: Coverage of 95% confidence intervals for between-study variance of LOR (the “only” versions of PL, QP and FPC; KD; and the model versions of FPU) vs τ^2, for equal sample sizes $n = 20,\ 40,\ 100$ and 250, $p_{iC} = .5$, $\theta = 0$ and $f = 0.5$.

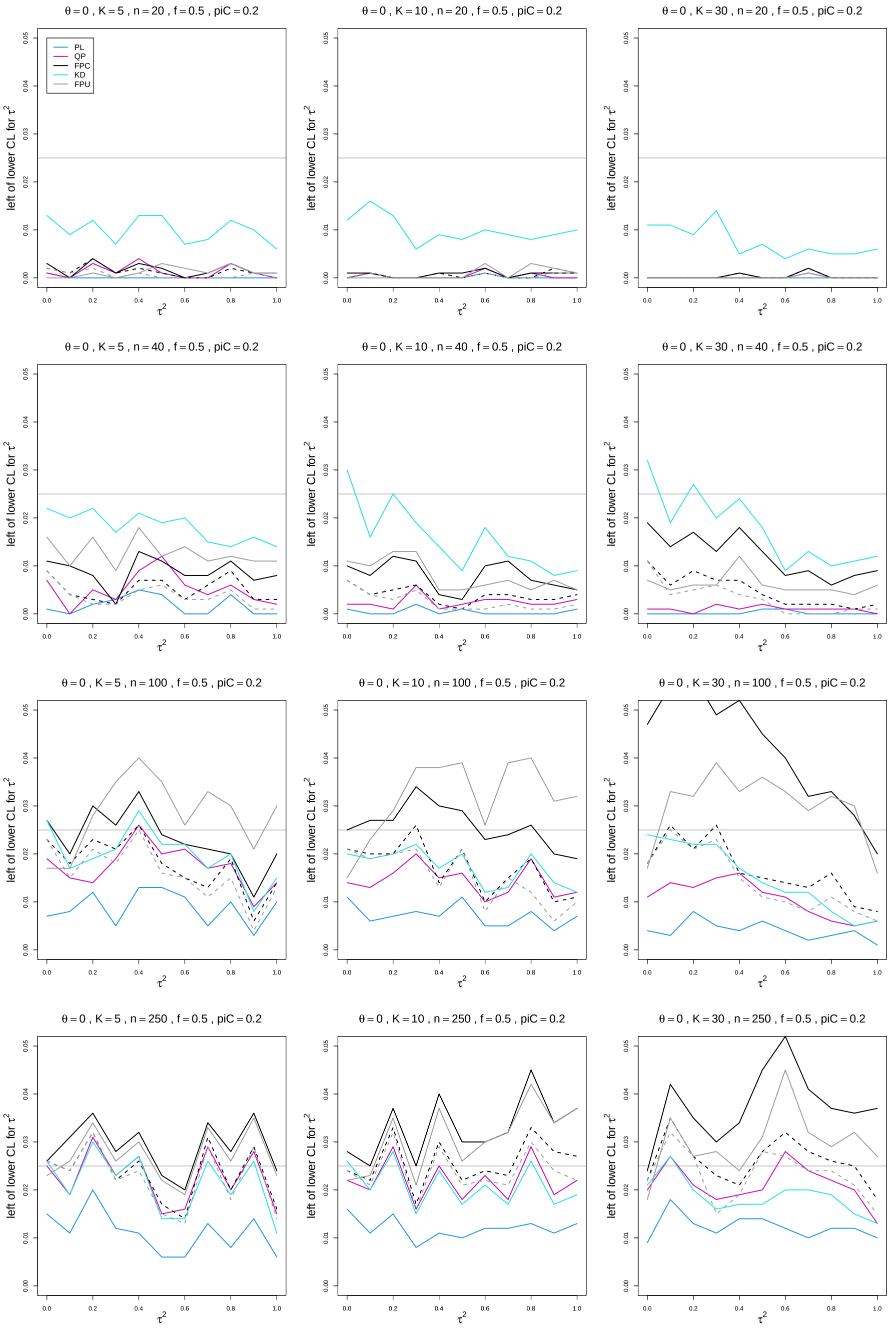

Figure S7: Miss-left probability of PL, QP, KD, FPC, and FPU 95% confidence intervals for between-study variance of LOR vs τ^2, for equal sample sizes n = 20, 40, 100 and 250, $p_{iC} = .2$, $\theta = 0$ and $f = 0.5$. Solid lines: the “only” versions of PL, QP and FPC; KD; and the model version of FPU. Dashed lines: the “always” version of FPC and the naïve version of FPU.

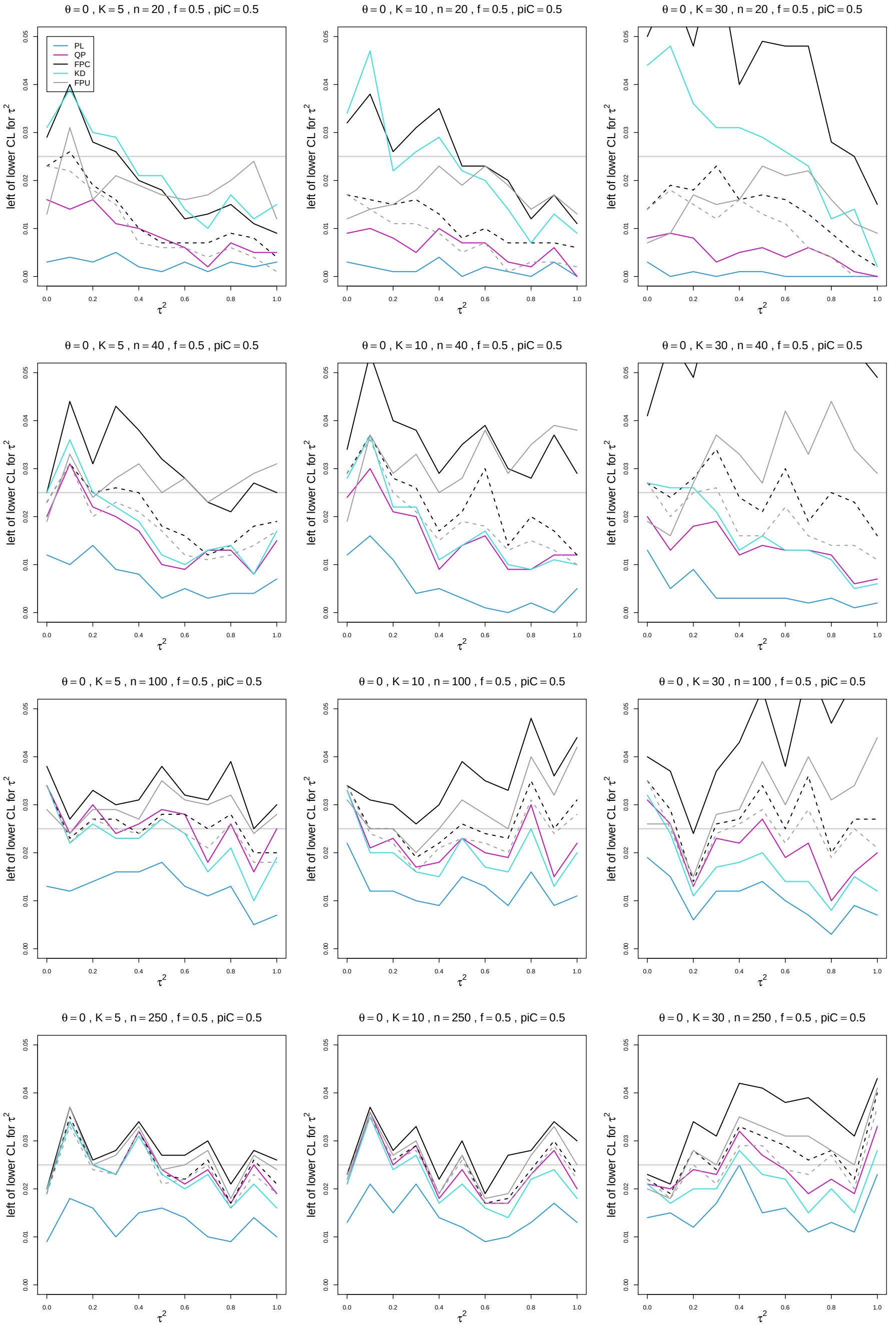

Figure S8: Miss-left probability of PL, QP, KD, FPC, and FPU 95% confidence intervals for between-study variance of LOR vs τ^2, for equal sample sizes $n = 20,\ 40,\ 100$ and 250, $p_{iC} = .5$, $\theta = 0$ and $f = 0.5$. Solid lines: the "only" versions of PL, QP and FPC; KD; and the model version of FPU. Dashed lines: the "always" version of FPC and the naïve version of FPU.

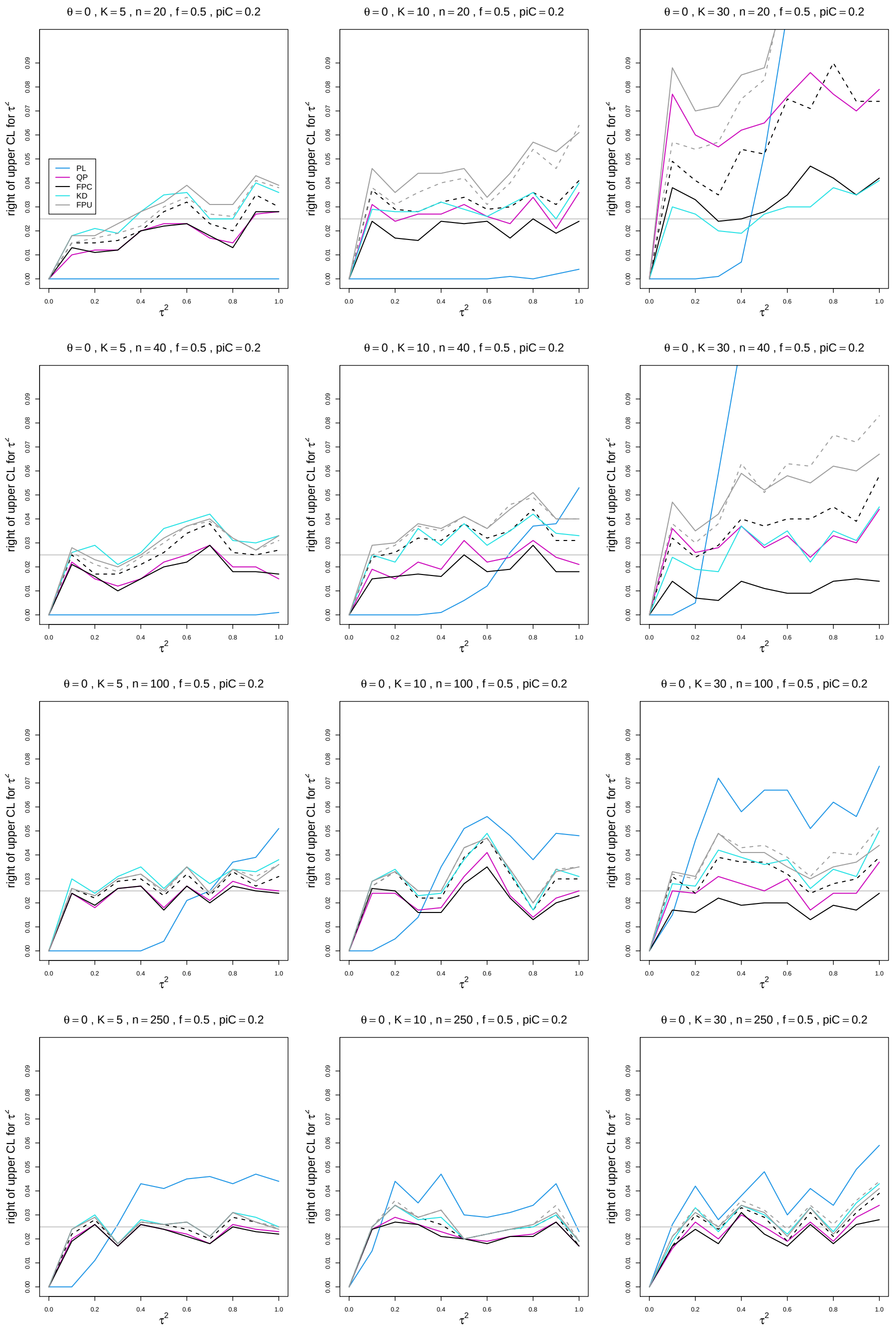

Figure S9: Miss-right probability of PL, QP, KD, FPC, and FPU 95% confidence intervals for between-study variance of LOR vs τ^2, for equal sample sizes $n = 20,\ 40,\ 100$ and 250, $p_{iC} = .2$, $\theta = 0$ and $f = 0.5$. Solid lines: the "only" versions of PL, QP and FPC; KD; and the model version of FPU. Dashed lines: the "always" version of FPC and the naïve version of FPU.

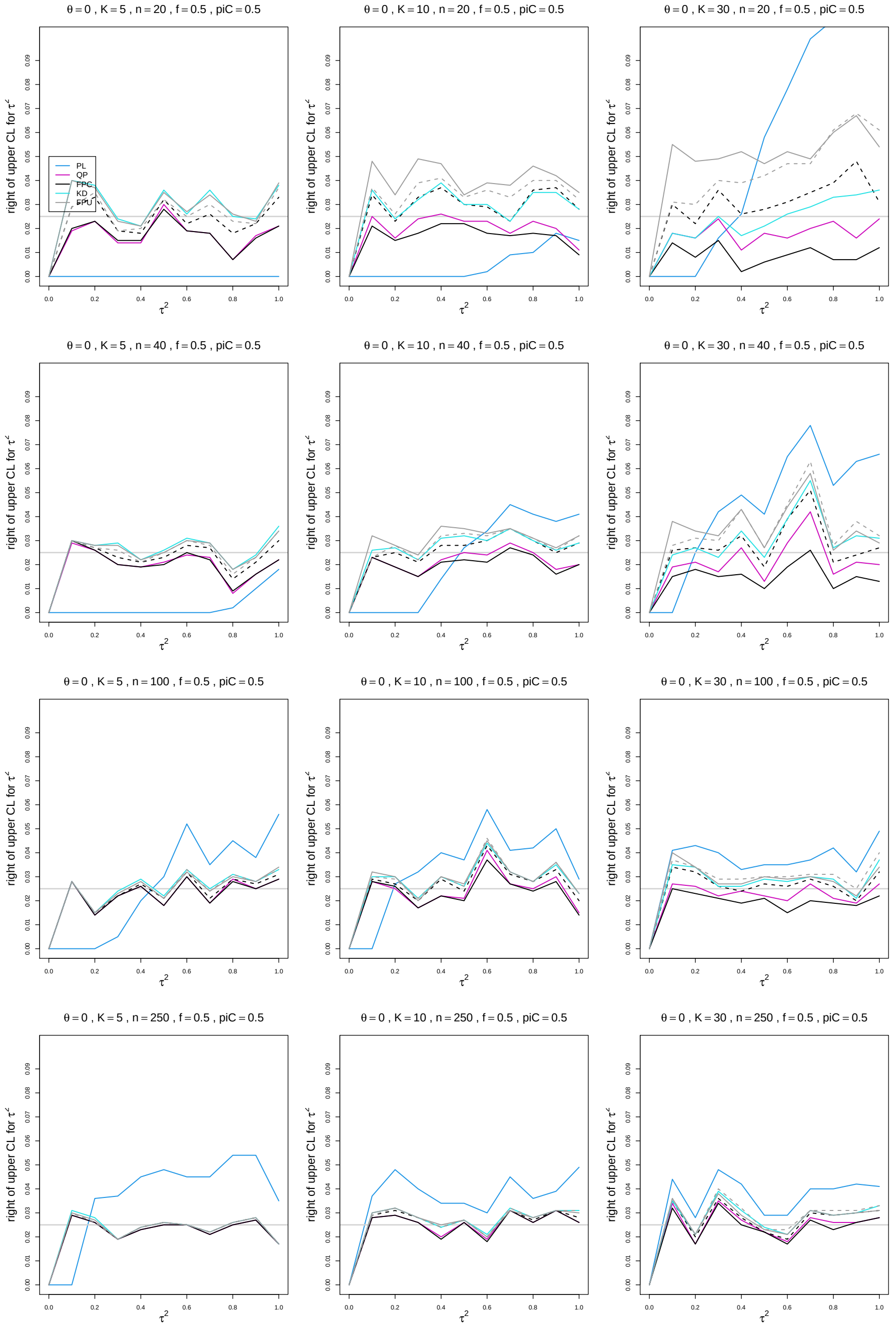

Figure S10: Miss-right probability of PL, QP, KD, FPC, and FPU 95% confidence intervals for between-study variance of LOR vs τ^2, for equal sample sizes $n = 20,\ 40,\ 100$ and 250, $p_{iC} = .5$, $\theta = 0$ and $f = 0.5$. Solid lines: the "only" versions of PL, QP and FPC; KD; and the model version of FPU. Dashed lines: the "always" version of FPC and the naïve version of FPU.

S3 Supplemental Tables

Table S1: Stead et al. (2013) data on the use of physician advice for smoking cessation

Study	X_T	n_T	X_C	n_C
Schnoll 2003	27	203	28	206
Vetter 1990	34	237	20	234
Higashi 1995	53	468	35	489
Russell 1979	34	1031	8	1107
Slama 1990	1	104	1	106
Janz 1987	26	144	12	106
Demers 1990	15	292	5	292
Stewart 1982	11	504	4	187
McDowell 1985	12	85	11	78
Wilson 1990	43	577	17	532
Russell 1983	43	740	35	637
Jamrozik 1984	77	512	58	549
Page 1986	8	114	5	68
Slama 1995	42	2199	5	929
Nebot 1989	11	208	5	216
Betson 1997	14	443	13	422
Porter 1972	5	101	4	90
Unrod 2007	28	237	18	228

Table S2: Summary statistics for selected estimators in simulations for Stead et al. example

θ	τ^2	p_{iC}	estimator	Min	Q1	Median	Mean	Q3	Max
			KD	0	0.007	0.039	0.056	0.089	0.404
			MP	0	0	0.029	0.048	0.075	0.392
0.5	0.05	X_{iC}/n_{iC}	SSC “always”	0	0	0.012	0.059	0.084	1.096
			SSU model	0	0	0.012	0.057	0.08	1.284
			SMC “only”	0	0	0.044	0.096	0.136	1.311
			KD	0	0.007	0.04	0.055	0.083	0.605
			MP	0	0.020	0.033	0.048	0.072	0.596
0.7	0.05	X_{iC}/n_{iC}	SSC “always”	0	0	0.012	0.063	0.092	1.090
			SSU model	0	0	0.011	0.061	0.090	1.014
			SMC “only”	0	0	0.043	0.101	0.145	1.400
			KD	0	0.115	0.184	0.202	0.269	0.87
			MP	0	0.104	0.169	0.187	0.252	0.843
0.5	0.2	X_{iC}/n_{iC}	SSC “always”	0	0.060	0.149	0.190	0.264	1.240
			SSU model	0	0.058	0.146	0.188	0.266	1.143
			SMC “only”	0	0.101	0.203	0.256	0.347	1.506
			KD	0	0.108	0.183	0.201	0.271	0.660
			MP	0	0.098	0.172	0.188	0.257	0.645
0.7	0.2	X_{iC}/n_{iC}	SSC “always”	0	0.063	0.145	0.190	0.274	1.395
			SSU model	0	0.065	0.142	0.187	0.271	1.400
			SMC “only”	0	0.101	0.198	0.252	0.348	1.648
			KD	0.010	0.136	0.194	0.200	0.251	0.559
			MP	0.010	0.136	0.194	0.200	0.251	0.559
0.5	0.2	$X_{iC}/n_{iC} + 0.1$	SSC “always”	0.015	0.128	0.184	0.199	0.252	0.692
			SSU model	0.015	0.129	0.184	0.198	0.252	0.691
			SMC “only”	0.019	0.143	0.204	0.22	0.279	0.758
